# Enhancement of Protein–Protein Interactions by Destabilizing Mutations Revealed by HDX-MS

**DOI:** 10.3390/biom15081201

**Published:** 2025-08-20

**Authors:** Yoshitomo Hamuro, Anthony Armstrong, Jeffrey Branson, Sheng-Jiun Wu, Richard Y.-C. Huang, Steven Jacobs

**Affiliations:** Johnson and Johnson Innovative Medicine, 1400 McKean Road, Spring House, PA 19477, USA; aarmst12@its.jnj.com (A.A.); jbranson@its.jnj.com (J.B.); swu4@its.jnj.com (S.-J.W.); rhuang35@its.jnj.com (R.Y.-C.H.); sjacobs9@its.jnj.com (S.J.)

**Keywords:** backbone amide, destabilization, hydrogen/deuterium exchange, mass spectrometry, mutation, protein–protein interaction

## Abstract

Enhancing protein–protein interactions is a key therapeutic strategy to ensure effective protein function in terms of pharmacokinetics and pharmacodynamics and can be accomplished with methods like directed evolution or rationale design. Previously, two papers suggested the possible enhancement of protein–protein binding affinity via destabilizing mutations. This paper reviews the results of the previous literature and adds new data to show the generality of the strategy that destabilizing the unbound protein without significantly changing the free energy of the complex can enhance protein–protein interactions for therapeutic benefit. The first example presented is that of a variant of human growth hormone (hGHv) containing 15 mutations that improve the binding to the hGH binding protein (hGHbp) by 400-fold while retaining full biological activity. The second example is that of the YTE mutations (M252Y/S354T/T256E) in the Fc region of a monoclonal antibody (mAb). The YTE mutations improve the binding of the mAb to FcRn at pH 6.0 10-fold, resulting in elongated serum half-life of the mAb. In both cases, (i) chemical titration or differential scanning calorimetry (DSC) showed the mutations destabilize the unbound mutant proteins, (ii) isothermal titration calorimetry (ITC) showed extremely favorable enthalpy (ΔH) and unfavorable entropy (ΔS) upon binding to their respective target molecule compared with the wildtype, and (iii) hydrogen/deuterium exchange–mass spectrometry (HDX-MS) revealed that these mutations increase the free energy of unbound mutant protein without significantly affecting the free energy of the bound state, resulting in an enhancement to the binding affinities. The third example presented is that of the JAWA mutations (T437R/K248E) also located in the Fc region of a mAb. The JAWA mutations facilitate antibody multimerization upon binding to cell surface antigens, allowing for enhanced agonism and effector functions. Both DSC and HDX-MS showed that the JAWA mutations destabilize the unbound Fc, although the complex was not characterized due to weak binding. Enhancement of protein–protein interactions through incorporation of mutations that increase the free energy of a protein’s unbound state represents an alternative route to decreasing the protein–protein complex free energy through optimization of the binding interface.

## 1. Introduction

Protein therapeutics constitute a fast-growing sector in the pharmaceutical industry with a market size approaching USD 400 billion [1]. There are many challenges faced by the pharmaceutical industry in generating protein therapeutics with better therapeutic properties, such as safety, efficacy, selectivity and stability, along with affordability and ease of administration. To optimize these therapeutic properties, various biophysical properties can be modulated, including aggregation propensity, colloidal and conformational stability, and binding affinities to the target and other interacting proteins [2,3].

The alteration of protein–protein interactions of therapeutic proteins leads to various therapeutic consequences. For example, improving the affinity to the target protein may lead not only to higher efficacy and higher selectivity, but also to better affordability by requiring a lower dose [4]. Modulating the affinity to proteins other than the target molecule may result in higher efficacy and safety through, for example, attenuated effector functions and better clearance/degradation properties [5].

Two general strategies to improve protein–protein interaction are directed evolution and rational design [6]. To obtain a protein with a certain property, directed evolution utilizes cycles of diversification of protein sequence and selection of resulting mutants with the desired property. Rational design, on the other hand, makes mutations to the protein sequence based on prior structural and functional knowledge. Since sequence–structure–function relationships are still difficult to predict accurately [6], evaluation of the results from directed evolution experiments can be used to inform rational design approaches.

In this regard, two papers suggested the possible enhancement of protein–protein binding affinity via destabilizing mutations [7,8]. This paper reviews the results of the previous literature and adds new data to show the generality of the strategy (Table 1). Overall, we present three examples in which improved protein–protein interactions were achieved through destabilization of the unbound protein with minimal changes to the free energy of the complex. Two of the examples, human growth hormone variant (hGHv) and the YTE mutant of immunoglobulin G (IgG), were identified through directed evolution (phage display). The third example, the JAWA mutant of IgG, was, in turn, rationally designed to facilitate antibody multimerization in part by destabilizing Fc.

## 2. Materials and Methods

### 2.1. Materials

All chemical reagents were obtained from Sigma-Aldrich (St. Louis, MO, USA) except the following items: Pierce^TM^ premium-grade Tris (2-carboxyethyl)phosphine hydrochloride (TCEP) was purchased from Thermo Fisher Scientific (Waltham, MA, USA), urea was purchased from VWR (Radnor, PA, USA), and water and acetonitrile were HPLC-grade from Honeywell (Charlotte, NC, USA).

### 2.2. Antibody Expression and Purification

The antibodies (WT, YTE and JAWA) were expressed and purified analogous to reference [11]. Plasmids encoding antibody HC and LC were co-transfected at a 1:3 (HC:LC) molar ratio into Expi293F cells following the transfection kit instructions (Thermo Fisher Scientific, Waltham, MA, USA). Cells were centrifuged 5 d post transfection and the supernatant was passed through a 0.2 mm filter. The titer of antibody expression was quantified using Octet (ForteBio, Fremont, CA, USA). Antibody purification was performed by affinity chromatography over MabSelect SuRe column (GE Healthcare Life Sciences, Chicago, IL, USA). The purified antibody was buffer-exchanged into Dulbecco’s phosphate-buffered saline (DPBS), pH 7.2, by dialysis, and protein concentration was determined by UV absorbance at 280 nm. Quality was assessed by SEC over a TSKgel G3SW column (Tosoh Bioscience, Grove City, OH, USA) in 1X DPBS pH 7.2 buffer and SDS-PAGE of reduced and non-reduced samples.

### 2.3. DSC

Thermal stability experiments were carried out in PBS. Antibodies were introduced into the sample chamber at concentrations of 1.0 mg/mL. DSC experiments were performed using a MicroCal Auto VP-capillary DSC system (Malvern Instruments Ltd., Malvern, UK) in which temperature differences between the reference and sample cell were continuously measured and converted to power units. The samples were heated from 25 °C to 110 °C at a rate of 1 °C/min. A pre-scan time of 10 min and a filtering period of 10 s were used for each run. DSC measurements were made at approximately 0.5 mL in PBS buffer in duplicate. Analysis of the resulting data was performed using MicroCal Origin software (version 7.0; MicroCal, Northampton, MA, USA).

### 2.4. ITC

All ITC experiments were conducted at 25 °C using an Affinity ITC Auto (Waters TA Instruments, New Castle, DE, USA). Human FcRn and IgG mAbs (wildtype and YTE mutant) were dialyzed at 4 °C overnight in ITC buffer (1 × PBS, pH 6.0). FcRn protein solution prepared at 15 μM was loaded into the sample cell at an effective volume and then titrated with 100 μM mAb solutions via the syringe. Each titration experiment contained 9 injections at 3 μL per injection and these were spaced by 300 s to ensure a complete return to baseline. Control experiments were performed by loading only the ITC buffer in the sample cell and injecting the 100 μM antibody solutions. The heat of dilution from the control experiments was subtracted from the heat generated in the FcRn experiments. The resulting isotherms were analyzed with an independent fit model using the Nano Analyze software (version 4.0.2.0; Waters TA Instruments), leading to the calculation of *K_D_*, *n*, Δ*H*, Δ*S*, and Δ*G* values.

### 2.5. HDX-MS

HDX-MS analysis for hGHv mutant was described elsewhere [7]. HDX-MS analyses for YTE mutant and JAWA mutant were performed as follows.

Non-deuterated and on-exchange experiment for HDX-MS: The on-exchange reaction was initiated by mixing 4 μL of 15 μM mAb with 36 μL of H_2_O or deuterated buffer (D_2_O with 10 mM Tris, 150 mM NaCl, pH 8.0). The reaction mixture was incubated for 15, 50, 150, 500, 1500, 5000, and 15,000 s at 23 °C. The exchanged solution was quenched by the addition of 40 μL of chilled 8 M urea, 1 M TCEP, pH 3.0 (pH was adjusted with aqueous NaOH) and immediately analyzed.

Fully deuterated experiment for HDX-MS: A fully deuterated sample was prepared by incubating a mixture of 9 μL of mAb and 81 μL of 100 mM TCEP in D_2_O at 55 °C for 3 h. After the fully deuterated sample was cooled down to 23 °C, a 40 μL aliquot of 8 M urea, 1 M TCEP, pH 3.0 was added to 40 uL of the fully deuterated sample and immediately analyzed.

General procedure for HDX-MS data acquisition: HDX-MS analysis was performed using an automated HDx3 system (LEAP Technologies, Morrisville, NC, USA).

The columns and pump configuration were as follows: protease, pepsin/protease type XIII (protease from *Aspergillus saitoi*, type XIII) column (*w*/*w*, 1:1; 2.1 × 30 mm) (NovaBioAssays Inc., Woburn, MA, USA); trap column, Acquity UPLC BEH C18 VanGuard Pre-column (Waters, Milford, MA, USA); analytical column, Accucore Vanquish C18 (2.1 × 100 mm, 1.5 µm) (Thermo Fisher Scientific); and LC pump, Vanquish (Thermo Fisher Scientific). The loading pump (from the protease column to the trap column) was set at 600 μL/min with 0.1% formic acid in water. The gradient pump (from the trap column to the analytical column) was set from 8% to 33% acetonitrile in 0.1% aqueous formic acid in 20 min at 100 μL/min.

MS data acquisition: Mass spectrometric analyses were carried out using an LTQ^TM^ Orbitrap Fusion Lumos mass spectrometer (Thermo Fisher Scientific) with the capillary temperature at 275 °C, resolution 120,000, and an *m*/*z* range of 300–1500.

HDX-MS data extraction: BioPharma Finder 2.0 (Thermo Fisher Scientific) was used for the peptide identification of non-deuterated samples prior to the HDX experiments. HDExaminer version 2.4 (Sierra Analytics, Modesto, CA, USA) was used to extract centroid values from the MS raw data files for the HDX experiments.

The deuteration level, *D*%, at each segment at each time point was calculated using Equation (1) in Excel [12].

(1) $$D\%=\frac{M(ON)-M(ND)}{M(FD)-M(ND)}$$

where *M*(*ON*) is the centroid value of the on-exchanged experiment, *M*(*ND*) is the centroid value of the non-deuterated experiment, and *M*(*FD*) is the centroid value of the fully deuterated experiment. In this equation, the number of deuterium incorporated in the on-exchanged experiment was divided by the number of deuterium incorporated in the fully deuterated experiment.

The deuteration level, *D*%, of sub-localized segment was calculated using Equation (2).

(2) $$D\%=\frac{\left[{M\left(ON\right)}_{a}-{M\left(ND\right)}_{a}]-[{M\left(ON\right)}_{b}-{M(ND)}_{b}\right]}{\left[{M\left(FD\right)}_{a}-{M\left(ND\right)}_{a}\right]-[{M\left(FD\right)}_{b}-{M(ND)}_{b}]}$$

where *a* indicates the centroid value of the larger segment used for sub-localization (e.g., segment 1–10) and *b* indicates the centroid value of the smaller segment used for sub-localization (e.g., segment 1–9). In this equation, the number of deuteriums incorporated at the sub-localized segment (e.g., residue 10) in the on-exchanged experiment was divided by the number of deuteriums incorporated at the sub-localized segment in the fully deuterated experiment.

All experimental exchange times were converted into those at pH 7 and 23 °C [13].

HDX-MS Data Analysis. Excel was used to further process and present the data. All HDX data were fitted with a stretched exponential curve (Equation (3)).

(3) *D* (*t*) = 1 − exp [−(*k t*)*^B^*]

Each stretched exponential curve yields a protection factor (Equation (4)) [14].

(4) $$pf=\frac{{kb(log2.3)}^{1/Ba}}{{ka(log2.3)}^{1/Bb}}$$

where *a* and *b* indicate two different sets of stretched exponential curves.

(5) Δ*G* = −*RT* ln (*pf*)

Equation (5) can convert a *pf* to the free energy change.

Peptide and Segment. Throughout this paper, the HDX-MS data were presented for “segment”, not for “peptide”. During bottom–up HDX-MS analysis, an analyte protein is digested into peptides to improve data resolution. The deuteron attached at the first residue of each peptide will be lost because the amide group becomes an amino group [13,15,16]. Also, most of the deuteron attached to the second residue will be lost because it also exchanges significantly faster than the rest of the amide hydrogens [13,15,16]. Due to these issues, the observed deuterium incorporation in a peptide (e.g., peptide 1–10) is reported as the deuterium incorporation in the corresponding segment (e.g., segment 3–10).

## 3. Results

### 3.1. Human Growth Hormone Variant (hGHv)

**Figure 1:**
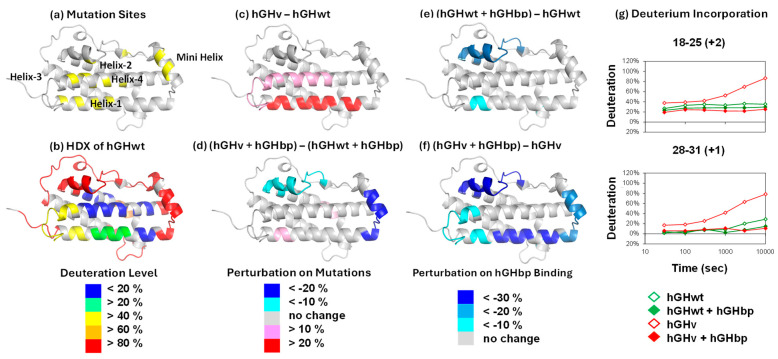
HDX-MS results of hGHwt and hGHv with X-ray crystal structure (PDB ID: 1HWH) [7]. (**a**) The mutation sites of hGHv are shown in yellow. They are F10A, M14W, H18D, H21N, K41I, Y42H, L45W, Q46W, F54P, R64K, R167N, D171S, E174S, F176Y, and I179T. (**b**) Average deuterium incorporation of each segment. The average deuteration level is color-coded. (**c**) The difference in deuterium incorporation between unbound hGHv and unbound hGHwt. (**d**) The difference in deuterium incorporation between hGHbp-bound hGHv and hGHbp-bound hGHwt. Red and pink indicate more deuterium incorporation and blue indicates less deuterium incorporation in hGHv than hGHwt for (**c**,**d**). (**e**) The perturbation of hGHwt upon binding to hGHbp. (**f**) The perturbation of hGHv upon binding to hGHbp. Blue indicates less deuterium incorporation upon binding to hGHbp for (**e**,**f**). (**g**) Deuterium incorporation in the selected segments of hGHwt and hGHv with and without hGHbp.

hGHv. A variant of human growth hormone (hGHv) generated through phage display mutagenesis binds to the extracellular domain of its cognate receptor human growth hormone binding protein (hGHbp) through Site-1 with a *K*_D_ < 10 pM, approximately 400-fold (3 kcal/mol) tighter compared to the wildtype hormone (hGHwt) [7,17]. hGHv contains 15 mutations in its Site-1 binding interface relative to hGHwt (Figure 1a) and retains its full biological activity.

Chemical Stability of hGHv. Guanidine chemical denaturation experiments of hGHwt and hGHv have previously been performed to understand the effects of the mutations introduced into hGHv on stability [7]. The stability of hGHwt was determined to be 11.6 kcal/mol, and that for hGHv was 8.0 kcal/mol (Table 2a,b) representing an approximately 3.5 kcal/mol decrease in stability of mutant hGHv relative to hGHwt.

Alanine Scanning of hGHv. Mutagenesis studies have shown that the functional epitope of hGHwt is characterized by a well-defined hot spot, where much of the binding energy comes from a small number of residues [18,19]. On the other hand, hGHv does not have a similar hot spot as hGHwt [20]. The new set of hydrogen bonds and van der Waals interactions introduced in hGHv do not appear to add significant binding energy to this interaction [20].

ITC of hGHv–hGHbp Interaction. One striking difference between the hGHwt-hGHbp interaction and the hGHv–hGHbp interaction is the distribution of enthalpic and entropic contributions to the binding thermodynamics (Table 3a,b) [9]. While the hGHwt-hGHbp interaction is characterized by favorable contributions from both enthalpic (Δ*H* = −9 kcal/mol) and entropic (−*T*Δ*S* = −3 kcal/mol) factors, the hGHv–hGHbp interaction is characterized by an extremely favorable enthalpy term (Δ*H* = −36 kcal/mol), counterbalanced by an unfavorable entropy component (−*T*Δ*S* = 21 kcal/mol) with the net effect being a decrease in the free energy of binding (ΔΔG = −3 kcal/mol).

X-Ray Crystallography of the hGHv–hGHbp Interaction. X-ray crystallographic data show that the extent of reorganization upon complex formation is mostly similar for both hGHwt and hGHv [7,21]. The discrepancy in thermodynamic parameters upon binding and the structural similarity between the hGHwt–hGHbp and hGHv–hGHbp complexes suggest that the structures of the unbound and bound molecules cannot explain the underlying mechanisms of the two binding interactions.

HDX-MS of Unbound hGHwt. The deuterium incorporation in each segment of unbound hGHwt [7] is consistent with the crystal structure of its G120R mutant (PDB ID: 1HWH). The four helix bundle regions of the protein exchanged slowly, whereas the loop regions exchanged fast (Figure 1b).

HDX-MS of Unbound hGHv. Some parts of hGHv exchanged faster than the corresponding hGHwt segments (Figure 1c,g), although the deuterium incorporation pattern of hGHv was like that of hGHwt in general (Figure S1 in Supplementary Materials) [7]. Overall, the similarity of deuterium incorporation patterns in hGHv and hGHwt is consistent with their structural similarity. On the other hand, the exchange rates of helix-1 of hGHv (segments 12–15, 18–25, and 28–31) were faster than those of hGHwt, and those of helix-4 (segments 166–176 and 179–191) were marginally so (Figure 1c; Figure S1 in Supplementary Materials), implying these protein–protein interface regions are more dynamic in hGHv.

HDX-MS of hGHwt and hGHv in the Presence of hGHbp. There were relatively small differences between deuterium incorporations in hGHwt and hGHv in the presence of hGHbp (Figure 1d; Figure S1 in Supplementary Materials) [7]. The only large difference in perturbation between hGHv and hGHwt in the presence of hGHbp was observed in the minihelix (residues 33–44; Figure 1d; Figure S1 in Supplementary Materials). The deuterium incorporations in helix-1 and helix-4, which exhibited a faster exchange in unbound hGHv compared to unbound hGHwt, showed no large difference when bound to hGHbp, suggesting that the complexes for hGHv and hGHwt had similar backbone energetics.

Binding to hGHbp decreased the deuterium incorporation in seven segments of hGHv, while only three segments were protected in hGHwt upon binding to hGHbp (Figure 1e,f; Figure S1 in Supplementary Materials) [7]. Among the four extra segments protected only in hGHv, helix-1 (segments 18–25 and 28–31) and helix-4 (segment 179–191) were protected because unbound hGHv exchanged faster than unbound hGHwt (Figure 1c) while exchanging at similar rates in the presence of hGHbp (Figure 1d). On the other hand, the minihelix (segment 33–44) was protected only in hGHv because of lower deuterium incorporation in hGHv upon binding to hGHbp (Figure 1d).

### 3.2. YTE Mutant

Neonatal Fc Receptor (FcRn). FcRn is a protein involved in the recycling of IgGs and in transferring antibodies from mother to fetus. The modulation of FcRn and IgG interaction attracts great interest in the pharmaceutical industry since a tighter binding of IgG to FcRn at acidic pH prevents the IgG from being transferred to the lysosome for degradation, ultimately extending its half-life in the circulation [22].

YTE Mutant. The YTE mutations, M255Y/S257T/T259E, identified by phage display enhance the affinity of IgG toward FcRn [23]. The YTE mutant has approximately 10-fold tighter binding to FcRn at pH 6.0 and 4-fold longer serum half-life [23,24,25] without causing major structural changes compared to the wildtype IgG [26,27].

DSC of YTE Mutant. The YTE mutant is conformationally less stable and more prone to aggregation than the corresponding wildtype [10]. DSC analysis showed that T_m_1 (melting temperature 1) of the YTE mutant (64 °C) is 6 °C lower than that of the wildtype (70 °C), reflecting a reduced stability of CH2 (constant region 2 in heavy chain) of the YTE mutant (Table 2c,d). On the other hand, T_m_2 (83 °C) and T_m_3 (87 °C) of the wildtype and YTE mutant were identical, indicating that the stability of the CH3 (constant region 3 in heavy chain) and Fab (fragment antigen-binding) domains was not affected by the mutation. The YTE mutant was shown to have a higher aggregation propensity than the wildtype in an accelerated storage study using size exclusion chromatography (SEC) [10].

ITC of YTE Mutant–FcRn Interaction. The thermodynamics of both wildtype IgG and YTE mutant binding to FcRn were determined by ITC at pH 6.0. Analogous to hGHv and hGHwt interactions with hGHbp, the thermodynamic signature of binding of the YTE mutant–FcRn interaction was very different from that of the wildtype–FcRn interaction (Table 3c,d; Figure S2 in Supplementary Materials). While the wildtype–FcRn interaction has both favorable enthalpic (Δ*H* = −5.5 kcal/mol) and entropic (−*T*Δ*S =* −3.0 kcal/mol) contributions, the YTE mutant–FcRn interaction has an extremely favorable enthalpic contribution (Δ*H* = −12.0 kcal/mol) and unfavorable entropic contribution (−*T*Δ*S* = 2.6 kcal/mol), equating to a tighter binding for the YTE mutant (ΔΔ*G* = −0.9 kcal/mol).

HDX-MS of Unbound YTE Mutant. Prior to HDX-MS analysis, a peptide map with HDX-MS compatible conditions was generated, and the sequence coverage of wildtype Fc and YTE Fc was 100% (=249/249; Figure S3 in Supplementary Materials).

**Figure 2:**
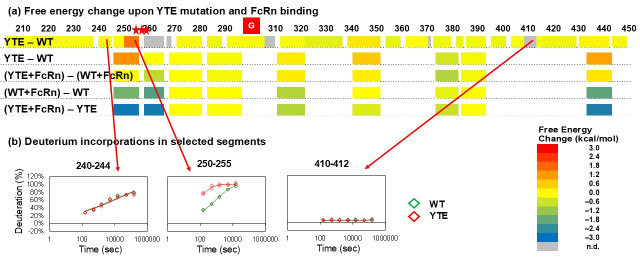
HDX-MS results of YTE mutant and the corresponding wildtype. (**a**) Free energy change in IgG Fc region upon YTE mutation and upon binding to FcRn. Red stars indicate YTE mutation sites. The top row is from the current study and the bottom four rows are from the work of Walters et al. [8]. The top two rows show the difference in free energy between unbound YTE mutant and unbound wildtype. The third row is the free energy difference in FcRn-bound proteins with and without YTE mutations. The fourth row is the free energy change in wildtype IgG upon binding to FcRn. The bottom row is the free energy change in the YTE mutant upon binding to FcRn. The energy change upon either YTE mutation or FcRn binding is color-coded as in the bottom right insert. (**b**) Deuterium incorporations in selected segments. Segment 240–244 was not perturbed. Segment 250–255 was destabilized upon YTE mutation. Segment 410–412 did not exchange at all and thus the perturbation was not observed. All exchange times are converted to those at pH 7 at 23 °C.

**Figure 3:**
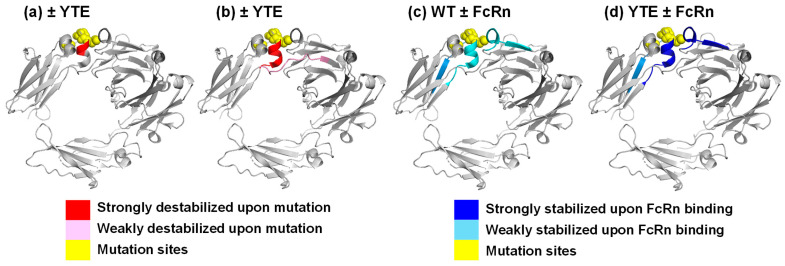
HDX-MS results of YTE mutant and the corresponding wildtype overlaid on X-ray crystal structure of Fc (PBD ID: 5VME). (**a**) Destabilized residues upon YTE mutation in the current study. (**b**) Destabilized residues upon YTE mutation in reference [8]. (**c**) Stabilized residues of wildtype upon FcRn binding in reference [8]. (**d**) Stabilized residues of the YTE mutant upon FcRn binding in reference [8].

Only one segment, residues 250–255, showed significant destabilization in the YTE mutant compared with wildtype (top row of Figure 2a; Figure 2b and Figure 3a,b; Table S1 and Figure S4 in Supplementary Materials). Previously, teams from the University of Kansas [10] and Genentech [8] also compared wildtype IgG versus YTE mutant using HDX-MS. Both saw analogous destabilizations in unbound YTE mutant. Overall, the HDX behaviors of the wildtype Fc and the YTE mutant Fc were similar to those typically observed for IgGs (Figure S5 in Supplementary Materials) and very similar to each other, indicating the YTE mutations do not alter the overall architecture of the Fc (Figure 2a; Figure S5 in Supplementary Materials).

The Genentech group went one step further by monitoring the HDX-MS of wildtype and YTE mutant in the presence of FcRn (bottom two rows of Figure 2a; Figure 3c,d; Table S2 in Supplementary Materials) [8]. The protection in the YTE mutant upon binding to FcRn is larger than that in the wildtype, because the unbound YTE mutant exchanged faster than the unbound wildtype, while bound YTE mutant and bound wildtype exchanged similarly.

### 3.3. JAWA Mutant

JAWA Mutant. JAWA mutations, T445R/K256E, were introduced into the IgG Fc to facilitate multimerization upon binding to cell surface antigens and enhance agonism [11]. While the T445R mutation was introduced with the intent of establishing a salt bridge interaction with E390 in a neighboring Fc upon dimerization, the K256E was introduced to facilitate multimerization by destabilizing the intramolecular CH2:CH3 interface, allowing the CH2 domain to more easily adopt conformations relative to CH3 compatible with a multimeric arrangement [28]. The double mutation was shown to have synergistic effects enhancing agonism [11].

DSC of JAWA Mutant. The JAWA mutant is conformationally less stable than the corresponding wildtype (Table 2e,f; Table S3 and Figure S6 in Supplementary Materials). DSC analysis showed relatively broad peaks for both the wildtype and the JAWA mutant, and three T_m_s were determined by deconvolution (Table 2e,f; Figure S6 in Supplementary Materials). The T_m_s for the wildtype were 73 °C (CH2), 77 °C (Fab) and 83 °C (CH3), while those for the JAWA mutant were 71 °C (CH2), 75 °C (CH3) and 77 °C (Fab). The JAWA mutations induced an 8 °C decrease in the CH3 domain stability, while only a 2 °C decrease was observed in the CH2 domain, and negligible change was observed in the Fab domain.

HDX-MS of JAWA Mutant. Prior to HDX-MS analysis, a peptide map with HDX-MS-compatible conditions was generated and the sequence coverage of both the wildtype and the JAWA mutant HC was 100% (=454/454), while that of the LC was 93% (193/220) (Figure S7 in Supplementary Materials).

**Figure 4:**
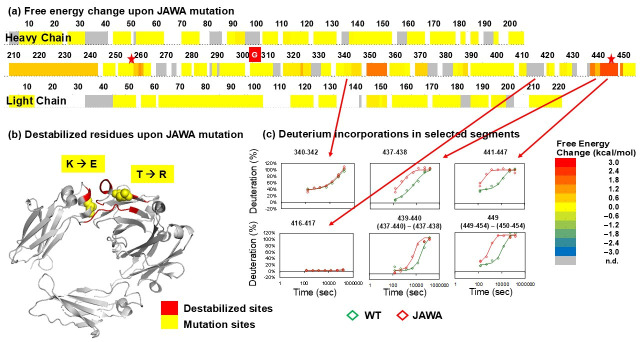
HDX-MS results of JAWA mutant (IgGB_JAWA_) and the corresponding wildtype (IgGB_WT_). (**a**) Difference in free energy between JAWA mutant and wildtype. Red stars indicate JAWA mutation sites. The energy change upon introduction of JAWA mutations is color-coded as in the bottom right insert. (**b**) Destabilized residues upon introduction of JAWA mutations determined in the current study overlaid on X-ray crystal structure of the JAWA mutant Fc (PBD ID: 5VME). (**c**) Segment 340–342 was not perturbed. Segment 416–417 did not exchange at all and thus the perturbation was not observed. Segments 437–438, 439–440, 441–447 and 449 were destabilized upon YTE mutation. All exchange times are converted to those at pH 7 at 23 °C.

In the JAWA mutant, the following residues near the mutation sites exchanged faster than in the wildtype, reflecting a destabilization in these regions: 259–260 (LM), 349–356 (GQPREPQV), 437–438 (HE), 441–447 (HNHYRQK), and 449 (L) (Figure 4; Figure S8 in Supplementary Materials). Overall, however, the HDX behaviors of the wildtype and JAWA mutant appeared consistent with those of a usual IgG (Figure S9 in Supplementary Materials) and similar to each other, indicating the JAWA mutations did not alter the general architecture of the IgG (Figure 4; Figure S9 in Supplementary Materials).

Fc domain-mediated multimerization can enhance agonism through clustering of cell surface receptors and similarly enhance effector functions [29,30]. IgGs with the JAWA mutations are monomeric in solution, suggesting a weak self-association [11]. Nonetheless, cell-based assays suggest a higher multimerization propensity for the JAWA mutant relative to wildtype upon binding target antigens [11]. Combining the designing rationale, DSC and HDX-MS of unbound molecules, as well as the cell-based assays, we believe that JAWA mutation enhances the oligomerization propensity of Fc at least partially by destabilizing the unbound state.

## 4. Discussion

Destabilizing Mutations. In all three examples discussed here, the mutants are shown to be destabilized relative to the wildtype proteins (Table 2). hGHv is observed to be destabilized by more than 3 kcal/mol in chemical denaturation experiments (Table 2a,b). The YTE mutations result in a 6 °C lower T_m_1, which corresponds to unfolding of the Fc CH2 domain (Table 2c,d), while the JAWA mutations result in an 8 °C reduction in the T_m_ of the CH3 domain and 2 °C for the CH2 domain (Table 2e,f).

HDX-MS analyses of the unbound states confirmed these conformational destabilizations and further sub-localized the areas affected by the mutations. hGHv has higher folding free energy in helices 1 and 4, where 9 out of 15 mutation sites are located (Figure 1c). Residues 250–255 of the YTE mutant showed destabilization compared with the corresponding wildtype (Figure 2 and Figure 3a). The destabilized residues are very close to the mutation sites, M255Y/S257T/T259E, in the CH2 domain, which showed a lower T_m_ in the mutant. The JAWA mutations induced destabilization in wide areas of the CH3 domain, residue 349–356, 437–438, 441–447 and 449, and in a smaller area of the CH2 domain, residues 259–260 (Figure 4). The two mutation sites, T445R/K256E, are within or near the destabilized areas. Destabilizations in wide areas of CH3 and in a smaller area of CH2 are consistent with a larger decrease in T_m_ for CH3 and a smaller decrease for CH2.

Enhancement of Protein–Protein Interactions by Destabilizing Mutations. SPR and ITC analyses showed that hGHv–hGHbp interaction and YTE Mutant–FcRn interaction have tighter dissociation constants than the corresponding wildtype interactions (Table 3). This means that the free energy decreases for the mutant interactions are larger than those for the wildtype interactions.

**Figure 5:**
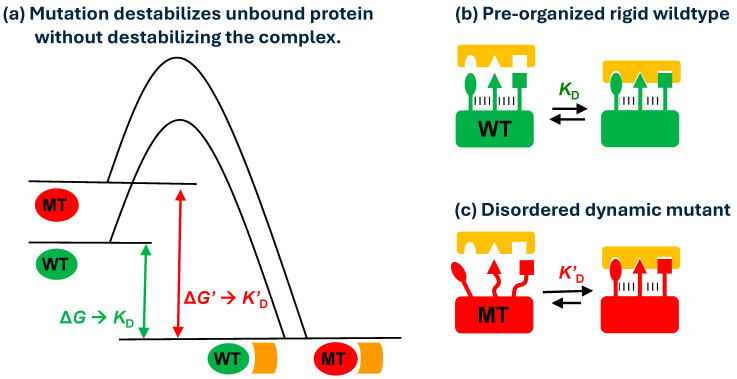
(**a**) Free energy diagram of wildtype (green) and destabilizing mutant (red). Destabilizing mutation gives higher free energy drop and smaller dissociation constant. (**b**) Binding of pre-organized wildtype (green) and its receptor (orange). Dotted line indicates hydrogen bonding. (**c**) Binding of disordered dynamic mutant (red) and its receptor (orange).

HDX-MS analyses showed relatively small free energy differences between hGHwt and hGHv when bound to hGHbp (Figure 1; Figure S1 in Supplementary Materials), as well as negligible free energy changes between a wildtype IgG and the corresponding YTE mutant when bound to FcRn (third row in Figure 2; Table S2 in Supplementary Materials). HDX-MS analyses of bound and unbound hGHv and IgG-YTE revealed that both mutation sets increased the free energy of the unbound states while having a smaller effect on the free energy the of bound states. These observations imply that the larger free energy decreases are primarily due to the increased free energy of the unbound states rather than decreased free energy of the bound states (Figure 5a).

Meaning of Protection Factor in HDX-MS Analysis and Destabilizing Mutants. Proteins are dynamic entities, and their backbone amide hydrogens participate in hydrogen bonding to varying degrees. The probability of involvement in hydrogen bonding is the biggest determinant of HDX rate [31]. Simplistically, if an amide hydrogen is involved in hydrogen bonding 99% of the time, the amide is accessible for the HDX reaction only 1% of the time, and the HDX rate may be 1% of the intrinsic exchange rate (protection factor = 100). In this regard, faster HDX rates for a mutant relative to wildtype in some backbone amide hydrogens in an unbound state indicate that the unbound mutant has a “looser” hydrogen bonding network than the unbound wildtype in the faster exchanging areas (Figure 5b,c).

In the YTE mutant example, the protection factor of segment 250–255 of the wildtype IgG was ~1,600 (Figure 2b; Table S1 in Supplementary Materials). This means 99.94% of the time the backbone amide hydrogens are participating in hydrogens and only 0.06% of the time are free to exchange, assuming the protection derives primarily from hydrogen bonding. The protection factor of this segment in the IgG YTE mutant was ~100 (Table S1 in Supplementary Materials), indicating 99% of the time hydrogen bonded and 1% of the time free. Although this segment is involved in hydrogen bonding in the ground state in both proteins, the hydrogen bonds in the mutant have a probability of being broken 16 times higher, and thus the hydrogen bonding network of the mutant is considered “looser” in this segment. The hGHv, IgG YTE, and IgG JAWA mutants all have similar structures to the corresponding wildtypes yet are more dynamic in certain areas [11,27,32].

Binding of Destabilizing Mutants. ITC analyses showed that both the hGHv–hGHbp interaction and YTE mutant–FcRn interaction have extremely favorable enthalpy contributions and unfavorable entropy contributions (Table 3). These unbalanced enthalpy/entropy contributions contrast with the thermodynamic signatures in the respective wildtype systems in which both enthalpy and entropy contribute favorably to binding (Table 3).

From the data presented here, we illustrate one strategy for enhancing protein–protein interactions, namely, through the introduction of mutations that destabilize the unbound state of one of the interacting proteins yet have a limited or neutral impact on the free energy of the complex (Figure 5b,c). Chemical denaturation and DSC studies (Table 2), as well as HDX-MS experiments performed in the absence of a binding partner, showed that the mutant proteins are destabilized relative to wildtype (Figure 1c, Figure 3a,b, Figure 4a and Figure 5b,c). In contrast, the HDX-MS experiments revealed mutant and wildtype proteins to have similar dynamic behavior in the bound state (Figure 5 b,c). This model is also consistent with the results from ITC, which showed increased enthalpy gains and unfavorable entropy losses upon binding of the mutant protein relative to the corresponding wildtype. In the model proposed in Figure 5c, the enthalpy gains in the case of mutant binding could result from the formation of new hydrogen bonds, while the entropy losses stem from restrictions imposed by freezing some conformational dynamics compared with the wildtype model in Figure 5b. It is worth pointing out that the nature of enhanced binding events by destabilizing mutations became apparent not by the analysis of static structures but by the analysis of dynamic properties using HDX-MS.

## 5. Conclusions

Here, three example systems were presented, illustrating one strategy to enhance protein–protein interactions by destabilizing a protein’s unbound state without significantly altering the free energy of the bound state. In two examples, those pertaining to the hGHv and IgG YTE mutants, mutations were identified through directed evolution approaches, whereas the IgG JAWA mutations in the third example were the result of rational design. In all cases, the mutants have lower stability relative to wildtype, as determined by either chemical denaturation studies or DSC, and are more dynamic in the unbound state, as determined by HDX-MS, while maintaining structures similar to that of wildtype. HDX-MS further revealed that both hGHv and IgG YTE mutants have similar dynamic properties to the respective wildtype molecules in the bound state. Consistently, in contrast to the wildtype proteins which bound with favorable entropy and enthalpy contributions, binding of the mutant proteins was characterized by very favorable enthalpy and unfavorable entropy contributions. Taken together, these data suggest the tighter affinities of the mutant proteins derive primarily from increased free energies of the unbound states rather than decreased free energies of the bound states.

## Figures and Tables

**Table 1:** Summary of experiments performed in the literature and the current study.

Protein	Chemical Stability	DSC	ITC	HDX-MS –Ligands	HDX-MS +Ligands
(a) hGHv	[7]	-----	[9]	[7]	[7]
(b) IgG-YTE	-----	[10]	current	[8], current	[8]
(c) IgGB-JAWA	-----	current	-----	current	-----

**Table 2:** Chemical and thermal stability of proteins.

Protein	Chemical Stability		DSC	
		CH2	CH3	Fab
(a) hGHwt *^1^	11.6 kcal/mol	-----	-----	-----
(b) hGHv *^1^	8.0 kcal/mol	-----	-----	-----
(c) IgGwt *^2^	-----	70 °C	83 °C	87 °C
(d) IgG-YTE *^2^	-----	64 °C	83 °C	87 °C
(e) IgG_B_wt	-----	73 °C	83 °C	77 °C
(f) IgG_B_-JAWA	-----	71 °C	75 °C	77 °C

*^1^ From reference [7]. *^2^ From reference [10].

**Table 3:** Dissociation constant and free energy of each protein–protein interaction.

Protein–Protein Interaction	SPR K_D_ (nM)	ΔG (kcal/mol)	ΔH (kcal/mol)	−TΔS (kcal/mol)
(a) hGHwt + hGHbp *^1^	<0.01	−12	−9	−3
(b) hGHv + hGHbp *^1^	~1.2	−15	−36	21
(c) IgG_A_wt + FcRn	5 *^2^	−8.4	−5.5	−3.0
(d) IgG_A_-YTE + FcRn	0.4 *^2^	−9.4	−12.0	2.6

*^1^ From reference [7]. *^2^ From reference [8].

## Data Availability

The original contributions presented in this study are included in this article/Supplementary Materials. Further inquiries can be directed to the corresponding author.

## Supplementary Materials

The following supporting information can be downloaded at: https://www.mdpi.com/article/10.3390/biom15081201/s1, Figure S1: Deuterium incorporation of hGHwt and hGHv with or without hGHbp; Figure S2: ITC of wildtype and YTE mutant against FcRn; Figure S3: A peptide map of WT and YTE mutant heavy chain digested by pepsin/FPXIII mixed bed column after quenched with 8 M urea, 1 M TCEP, pH 3.0; Figure S4: HDX-MS results of WT and YTE mutant heavy chains; Figure S5: Folding free energy of WT mAb Fc; Figure S6: DSC of WT mAb and JAWA mutant mAb; Figure S7: A peptide map of WT mAb and JAWA mutant mAb digested by pepsin/FPXIII mixed bed column after quenched with 8 M urea, 1 M TCEP, pH 3.0; Figure S8: HDX-MS results of WT and JAWA mutant; Figure S9: Folding free energy of WT mAb; Table S1: Folding free energy of WT mAb Fc and change in folding free energy upon YTE mutation (ΔΔG, kcal/mol); Table S2: Change in folding free energy upon YTE mutation and FcRn binding (ΔΔG, kcal/mol); Table S3: Folding free energy of WT mAb (ΔG, kcal/mol) and change in folding free energy between WT mAb and JAWA mutant mAb (ΔΔG, kcal/mol).

## Abbreviations

CH2, constant region 2 in heavy chain; CH3, constant region 3 in heavy chain; DPBS, Dulbecco’s phosphate-buffered saline; DSC, differential scanning calorimetry; Fab, fragment antigen-binding; Fc, fragment crystallizable; FcRn, neonatal Fc receptor for IgG; HC, heavy chain; HDX, hydrogen/deuterium exchange; hGH, human growth hormone; hGHbp, extracellular domain of human growth hormone cognate receptor; IgG, immunoglobulin G; ITC, isothermal titration calorimetry; JAWA mutations, K256E/T445R; mAb, monoclonal antibody; LC, light chain; MS, mass spectrometry; pf, protection factor; PPI, protein–protein interaction; SEC, size exclusion chromatography; SPR, surface plasmon resonance; TCEP, Tris(2-carboxyethyl)phosphine hydrochloride; T_m_, melting temperature; Tris, tris(hydroxymethyl)aminomethane; YTE mutations, M252Y/S354T/T256E.
