# Supplementary material for: Enhancement of Protein–Protein Interactions by Destabilizing Mutations Revealed by HDX-MS
Source: Biomolecules. 2025 Aug 20;15(8):1201. doi: 10.3390/biom15081201 (PMC12384571; doi:10.3390/biom15081201)
Supplement: Supplementary file 1 [file biomolecules-15-01201-s001.zip › biomolecules-3733364-supplementary.pdf]

Enhancement of Protein-Protein Interactions by Destabilizing Mutations Revealed by HDX-MS.

Yoshitomo Hamuro *, Jeffrey Branson, Sheng-Jiun Wu, Anthony Armstrong, Richard Huang and Steven Jacobs

Johnson and Johnson Innovative Medicine, 1400 McKean Road, Spring House, PA 19477, USA

* Corresponding author: yhamuro@its.jnj.com

Supporting Information

List of Tables

Table S1. Folding free energy of WT mAb Fc and change in folding free energy upon YTE mutation (ΔΔG, kcal/mol).

Table S2. Change in folding free energy upon YTE mutation and FcRn binding (ΔΔG, kcal/mol).

Table S3. Folding free energy of WT mAb (ΔG, kcal/mol) and change in folding free energy between WT mAb and JAWA mutant mAb (ΔΔG, kcal/mol).

List of Figures

Figure S1. Deuterium incorporation of hGHwt and hGHv with or without hGHbp. Each panel represents a segment of the protein. The title shows the residue numbers in each segment. The number in the parenthesis is the charge state of the peptide analyzed.

Figure S2. ITC of wildtype and YTE mutant against FcRn. Left panel, the integrated peaks are represented by a fitted curve. Right panel, the raw data with integrated baseline.

Figure S3. A peptide map of WT and YTE mutant heavy chain digested by pepsin / FPXIII mixed bed column after quenched with 8 M urea, 1 M TCEP, pH 3.0. G indicates glycosylation site. **Red** stars are YTE mutation sites.

Figure S4. HDX-MS results of WT and YTE mutant heavy chains. Each panel represents deuterium buildup curves of each segment: **Green**, WT; and **red**, YTE. All exchange times are converted to those at pH 7 at 23 °C. White panels are the segments used in the analysis. Yellow panels are backups.

Figure S5. Folding free energy of WT mAb Fc. Reddish color indicates unstable residue (usually not hydrogen bonded) and blueish color indicates stable residue (usually hydrogen bonded). The residues without color indicate the HDX behaviors were not monitored, because there is no peptide to cover the residues or the residues are the first two residues of a peptide. G indicates glycosylation site. **Red** stars are YTE mutation sites.

Figure S6. DSC of WT mAb and JAWA mutant mAb.

Figure S7. A peptide map of WT mAb and JAWA mutant mAb digested by pepsin / FPXIII mixed bed column after quenched with 8 M urea, 1 M TCEP, pH 3.0. G indicates glycosylation site. **Red** stars are JAWA mutation sites.

Figure S8a. HDX-MS results of WT and JAWA mutant heavy chains. Each panel represents deuterium buildup curves of WT and JAWA mutant segment: **Green**, WT; **red**, JAWA mutant. All exchange times are converted to those at pH 7 at 23 °C. White panels are the segments used in the analysis. Yellow panels are backups.

Figure S8b. HDX-MS results of WT and JAWA mutant light chains. Each panel represents deuterium buildup curves of WT and JAWA mutant segment: **Green**, WT; **red**, JAWA mutant. All exchange times are converted to those at pH 7 at 23 °C. White panels are the segments used in the analysis. Yellow panels are backups.

Figure S9. Folding free energy of WT mAb. Reddish color indicates unstable residue (usually not hydrogen bonded) and blueish color indicates stable residue (usually hydrogen bonded). The residues without color indicate the HDX behaviors were not monitored, because there is no peptide to cover the residues or the residues are the first two residues of a peptide. **Red** residues are the mutation sites between WT and JAWA mutant. **G** indicates glycosylation site. **Red** stars are JAWA mutation sites.

Table S1. Protection factor of WT mAb Fc, protection factor of YTE mAb Fc, folding free energy of WT mAb Fc (ΔG, kcal/mol) and change in folding free energy upon YTE mutation (ΔΔG, kcal/mol).

start	end	charge	pf (WT)	pf (YTE)	ΔG (WT)	ΔΔG (YTE)
203	237	6	9575	11334	-5.4	-0.1
240	243	1	1419	1634	-4.3	-0.1
240	244	1	6656	8319	-5.2	-0.1
240	255	2	3729	1273	-4.8	0.6
246	251	3	11690	54750	-5.5	-0.9
246	254	3	1608	88	-4.3	1.7
246	255	3	1616	279	-4.3	1.0
247	251	2	318	1103	-3.4	-0.7
247	254	3	1427	23	-4.3	2.4
247	255	3	963	78	-4.0	1.5
250	255	1	1554	98	-4.3	1.6
258	263	2	66293	80448	-6.5	-0.1
258	264	2	38236	42837	-6.2	-0.1
258	265	2	3829395	1155723	-7.7	NDslow
267	268	1	1371921	1617938	-7.7	NDslow
267	280	3	3679	4279	-4.8	-0.1
268	280	3	1856	2094	-4.4	-0.1
271	280	3	1101	1311	-4.1	-0.1
283	288	2	19	26	-1.7	-0.2
283	303	4	118	107	-2.8	0.1
283	308	5	151	216	-3.0	-0.2
283	309	4	335	421	-3.4	-0.1
291	305	0	122	134	-2.8	-0.1
291	309	4	774	978	-3.9	-0.1
306	309	1	757438	848290	-7.7	NDslow
312	321	3	34023	39630	-6.1	-0.1
312	336	5	23003	29799	-5.9	-0.2
312	351	5	8765	11663	-5.3	-0.2
322	336	0	9632	16297	-5.4	-0.3
339	351	4	212	128	-3.2	0.3
339	368	5	119	152	-2.8	-0.1
354	359	2	346	545	-3.4	-0.3
354	368	3	75	125	-2.5	-0.3
354	370	3	13	20	-1.5	-0.2
362	368	2	0	0	0.4	0.0
362	370	2	6	2	-1.0	0.5
362	371	2	23551	84283	-5.9	-0.7
371	371	1	22000000	22000000	-7.7	NDslow
371	383	2	143249	142223	-7.0	0.0
373	379	2	37785	49806	-6.2	-0.2
373	381	2	55265	65856	-6.4	-0.1
373	383	2	21401	23281	-5.9	0.0

373	384	2	31530	37439	-6.1	-0.1
382	384	1	2150	3289	-4.5	-0.2
386	393	1	68	83	-2.5	-0.1
386	401	2	58	85	-2.4	-0.2
386	407	2	155	245	-3.0	-0.3
396	401	2	104318	154184	-6.8	-0.2
404	408	1	50597	48184	-6.4	0.0
404	409	1	3518561	6007803	-7.7	NDslow
409	409	1	22000000	22000000	-7.7	NDslow
410	412	2	22000000	22000000	-7.7	NDslow
410	413	2	22000000	22000000	-7.7	NDslow
410	417	3	1598578	1949236	-7.7	NDslow
411	413	2	22000000	22000000	-7.7	NDslow
412	413	1	22000000	22000000	-7.7	NDslow
412	417	2	36112	41944	-6.2	-0.1
414	417	0	1975	1925	-4.5	0.0
416	425	2	171	170	-3.0	0.0
416	426	3	418	510	-3.6	-0.1
416	428	3	2097	2459	-4.5	-0.1
416	429	3	3571	4964	-4.8	-0.2
431	442	4	18469	14878	-5.8	0.1
431	449	4	1476	1359	-4.3	0.0
432	442	4	30752	25483	-6.1	0.1
432	449	3	1368	1551	-4.2	-0.1
444	449	2	36	44	-2.1	-0.1

ΔG is folding free energy of WT mAb Fc of each segment (corresponding to Figure S5). ΔΔG is the difference in free energy change upon YTE mutation of each segment (corresponding to Figure 2a). NDslow indicates the segment exchanged too slow to determine the ΔΔG. White lines are the segments used in Figures 2 and S5. Yellow lines are backups and correspond to the yellow panels in Figure S5.

Table S2. Change in folding free energy upon YTE mutation and FcRn binding (ΔΔG, kcal/mol).*

start	end	ΔΔG (WT±YTE)	ΔΔG (WT+FcRn±YTE)	ΔΔG (WT±FcRn)	ΔΔG (YTE±FcRn)
243	252	1.3	0.1	-1.6	-2.8
255	262	0.0	-0.7	-2.0	-2.8
265	277	0.0	0.0	0.0	0.0
280	293	-0.1	-0.1	0.0	0.0
308	318	0.2	0.2	-0.9	-0.9
338	348	0.6	0.2	0.0	-0.4
371	379	-0.5	-0.9	-0.3	-0.6
381	390	-0.1	-0.1	0.0	0.0
431	440	1.1	0.3	-1.9	-2.6

* Calculated from Walters et al., J. Biol. Chem., 2016, 1817-1825 using Eqs. 3 – 5 in the main text.

Table S3. Folding free energy of WT mAb (ΔG, kcal/mol) and change in folding free energy between WT mAb and JAWA mutant mAb (ΔΔG, kcal/mol). Folding free energy of JAWA mAb Fc is ΔG + ΔΔG.

chain	start	end	charge	ΔG	ΔΔG
HC	3	6	1	-7.7	NDslow
HC	3	14	2	-1.8	-0.1
HC	3	17	2	-1.8	-0.1
HC	3	20	2	-1.7	-0.2
HC	3	23	3	-2.7	-0.1
HC	7	14	0	-3.5	-0.1
HC	15	20	0	-2.7	-0.5
HC	21	23	0	-5.9	0.0
HC	26	32	1	-3.2	-0.1
HC	26	37	2	-2.9	0.0
HC	33	37	0	-7.7	NDslow
HC	38	40	2	-7.7	NDslow
HC	38	41	2	-7.7	NDslow
HC	38	50	3	-3.4	-0.1
HC	41	41	0	-7.7	NDslow
HC	44	49	2	-3.3	-0.1
HC	44	50	2	-3.3	-0.1
HC	49	50	1	-3.3	-0.1
HC	52	52	1	-7.7	NDslow
HC	52	54	2	-7.7	NDslow
HC	53	55	1	-7.6	0.0
HC	53	56	2	-6.4	-0.1
HC	56	56	0	-5.0	0.0
HC	57	62	3	-3.2	0.0
HC	57	63	3	-3.0	-0.1
HC	57	70	4	0.3	-0.4
HC	64	70	0	0.4	0.0

HC	65	67	1	-1.8	0.0
HC	70	72	0	-4.4	0.0
HC	70	82	3	-0.5	-0.1
HC	70	86	4	-4.7	-0.1
HC	73	77	0	-3.1	-0.1
HC	73	82	2	0.4	0.0
HC	73	86	2	-4.6	0.0
HC	78	82	2	0.4	0.0
HC	78	86	2	-7.7	NDslow
HC	82	84	0	-7.7	NDslow
HC	82	86	2	-7.7	NDslow
HC	85	85	0	-7.7	NDslow
HC	85	86	1	-7.7	NDslow
HC	86	86	1	-7.7	NDslow
HC	89	91	1	-7.1	0.0
HC	89	96	2	-6.3	0.0
HC	89	98	1	-7.7	NDslow
HC	92	96	1	-6.3	0.2
HC	96	97	1	-7.7	NDslow
HC	96	98	1	-7.7	NDslow
HC	98	98	0	-7.7	NDslow
HC	99	101	2	-7.7	NDslow
HC	99	108	3	-2.5	-0.2
HC	103	103	0	-4.7	0.0
HC	103	108	2	-2.6	-0.2
HC	104	104	0	-3.4	-0.3
HC	104	108	2	-3.2	-0.1
HC	105	106	0	-2.8	-0.4
HC	105	108	2	-3.1	-0.1
HC	107	108	1	-3.1	-0.1
HC	111	113	0	-5.3	0.1
HC	111	120	2	-3.4	-0.1
HC	114	115	0	-2.8	-0.7
HC	114	120	1	-1.8	-0.3
HC	116	120	1	-3.7	0.0
HC	123	131	2	-4.3	0.0
HC	123	153	3	-3.8	0.0
HC	134	136	1	-7.7	NDslow
HC	137	138	1	-5.0	0.1
HC	139	153	2	-1.6	-0.2
HC	156	157	0	-7.7	NDslow
HC	156	159	2	-7.7	NDslow
HC	156	163	2	-5.3	0.0
HC	156	166	2	-3.7	-0.1
HC	158	159	1	-7.7	NDslow
HC	158	166	2	-3.1	-0.1

HC	159	166	2	-3.3	-0.2
HC	160	165	0	-3.5	-0.2
HC	160	166	2	-3.1	-0.2
HC	166	166	1	-3.2	-0.2
HC	169	173	0	-2.8	-0.6
HC	169	178	2	-1.3	-0.1
HC	169	182	2	-1.4	-0.2
HC	172	178	2	-1.9	-0.3
HC	174	178	2	-3.7	-0.1
HC	174	182	2	-7.7	NDslow
HC	181	182	1	-7.1	0.0
HC	181	187	1	-3.1	-0.1
HC	185	187	1	-5.2	0.1
HC	190	193	1	-7.7	NDslow
HC	193	195	0	-7.4	0.0
HC	193	201	1	-2.6	0.0
HC	193	205	2	0.4	0.0
HC	196	201	1	0.4	0.0
HC	196	205	2	-2.5	-0.3
HC	200	205	1	-2.2	-0.8
HC	208	242	5	-3.7	0.6
HC	211	242	5	-2.1	0.4
HC	245	246	1	-2.3	-0.7
HC	245	249	1	-3.6	-0.1
HC	247	249	0	-4.3	0.0
HC	251	253	0	-4.2	-0.1
HC	251	256	3	-2.8	-0.4
HC	251	260	2	-4.3	0.6
HC	254	256	1	-3.0	-0.3
HC	254	260	2	-4.2	0.5
HC	256	258	0	-4.3	0.6
HC	256	259	1	-4.3	0.9
HC	259	259	1	-4.8	1.2
HC	259	260	1	-4.6	1.1
HC	260	260	0	-4.6	1.0
HC	261	261	0	-3.2	-0.3
HC	261	263	2	-2.8	-0.2
HC	262	263	1	-2.9	-0.1
HC	263	268	2	-4.0	0.0
HC	263	269	2	-5.5	-0.1
HC	266	268	1	-7.7	NDslow
HC	266	269	1	-7.7	NDslow
HC	269	269	0	-7.7	NDslow
HC	272	273	1	-7.7	NDslow
HC	272	285	3	-3.6	0.0
HC	276	279	2	-2.7	-0.5

HC	276	285	3	-5.7	0.0
HC	281	283	2	-5.3	0.1
HC	281	285	2	-4.1	0.0
HC	283	284	0	-3.2	-0.4
HC	283	285	2	-4.9	-0.1
HC	285	285	1	-6.4	0.0
HC	287	287	1	-7.7	NDslow
HC	287	288	1	-3.7	-0.2
HC	287	289	1	-3.3	-0.1
HC	288	288	1	-3.6	-0.1
HC	288	289	1	-3.4	-0.1
HC	289	289	0	-3.2	-0.1
HC	290	290	0	-7.7	NDslow
HC	290	293	1	-2.3	-0.3
HC	291	293	1	-0.3	-0.1
HC	292	300	3	-2.8	-0.1
HC	293	300	3	-2.1	-0.4
HC	303	305	0	-3.4	-0.1
HC	303	308	2	-2.9	-0.2
HC	303	309	2	-2.4	-0.2
HC	305	309	2	-2.6	-0.2
HC	306	309	2	-3.0	-0.1
HC	310	311	0	-6.1	-0.3
HC	310	314	2	-6.9	-0.2
HC	311	314	2	-7.7	NDslow
HC	312	313	1	-7.6	0.0
HC	312	314	1	-7.7	NDslow
HC	316	320	2	-2.8	0.2
HC	317	330	5	-6.3	0.2
HC	317	334	4	-6.1	0.2
HC	320	322	0	-6.5	0.3
HC	320	330	4	-6.9	0.3
HC	323	323	1	-6.1	0.9
HC	323	326	2	-5.9	0.4
HC	323	330	3	-7.6	0.3
HC	324	326	0	-5.9	0.3
HC	327	330	0	-7.7	NDslow
HC	331	334	0	-4.4	-0.3
HC	337	339	0	-4.0	-0.1
HC	337	342	2	-5.7	0.1
HC	337	346	3	-5.3	0.3
HC	340	342	1	-7.2	0.1
HC	342	346	2	-5.5	0.4
HC	349	356	3	-1.5	1.6
HC	349	365	3	-2.1	0.6
HC	358	363	2	-1.9	0.1

HC	358	365	2	-2.3	-0.2
HC	364	365	0	-3.7	-0.4
HC	368	373	2	-2.7	0.5
HC	368	376	2	-7.7	NDslow
HC	373	375	0	-7.7	NDslow
HC	373	376	1	-7.7	NDslow
HC	376	376	1	-7.7	NDslow
HC	379	382	2	-7.7	NDslow
HC	379	384	2	-5.1	0.5
HC	379	386	2	-5.6	0.3
HC	379	388	2	-5.2	0.3
HC	383	384	0	-3.8	-0.2
HC	385	386	0	-6.7	0.1
HC	387	388	1	-5.2	0.4
HC	390	390	1	-7.3	-0.4
HC	390	406	2	-0.4	-0.6
HC	391	392	1	-3.3	-0.2
HC	391	398	1	-2.7	-0.2
HC	391	406	3	0.4	0.0
HC	393	398	0	-3.4	-0.1
HC	399	402	0	-2.8	-0.7
HC	399	406	2	-3.2	-0.6
HC	401	406	2	-3.5	-0.6
HC	402	406	2	-7.7	NDslow
HC	403	406	1	-3.9	-0.5
HC	409	412	1	-3.0	0.1
HC	409	413	1	-6.8	0.2
HC	409	414	1	-7.7	NDslow
HC	412	412	1	-7.7	NDslow
HC	412	413	1	-7.7	NDslow
HC	412	414	1	-7.7	NDslow
HC	413	413	0	-7.7	NDslow
HC	414	414	1	-7.7	NDslow
HC	415	415	1	-7.7	NDslow
HC	415	417	2	-7.7	NDslow
HC	415	418	2	-7.7	NDslow
HC	416	417	1	-7.7	NDslow
HC	417	417	1	-7.7	NDslow
HC	417	418	1	-7.7	NDslow
HC	417	422	3	-4.0	-0.1
HC	418	418	0	-7.7	NDslow
HC	420	420	0	-4.5	0.2
HC	420	422	1	-4.0	-0.2
HC	421	422	1	-3.9	-0.2
HC	421	431	3	-3.1	0.0
HC	421	433	3	-7.7	NDslow

HC	425	428	1	-4.2	0.2
HC	425	431	2	-4.2	0.1
HC	425	433	2	-7.7	NDslow
HC	428	429	0	-3.3	-0.3
HC	428	431	1	-7.7	NDslow
HC	430	431	1	-7.7	NDslow
HC	436	436	0	-7.7	NDslow
HC	436	438	1	-5.7	1.5
HC	437	438	1	-5.6	1.6
HC	437	440	2	-6.4	1.0
HC	439	440	0	-6.7	0.8
HC	440	440	1	-6.7	1.2
HC	440	447	2	-6.2	1.9
HC	441	447	3	-6.1	2.1
HC	449	449	0	-6.1	1.5
HC	449	454	2	-1.0	0.0
HC	450	452	0	-3.2	-0.2
HC	450	454	1	-4.2	0.0
HC	453	454	1	-3.3	-0.9
LC	3	12	2	0.4	-0.2
LC	33	43	0	-7.7	NDslow
LC	33	52	3	-7.1	-0.6
LC	44	52	3	-3.7	-0.3
LC	55	60	2	-4.8	-0.3
LC	55	76	3	-3.2	0.2
LC	62	102	5	-1.6	-0.4
LC	114	122	3	-3.3	-0.9
LC	124	131	2	-3.8	-0.5
LC	124	138	2	-2.7	-0.2
LC	124	141	2	-5.5	0.4
LC	125	128	1	-3.4	-0.1
LC	125	138	2	-2.5	-0.2
LC	125	141	2	-4.5	0.1
LC	129	130	0	-3.7	-0.3
LC	129	131	1	-3.7	-0.1
LC	129	138	2	-3.8	0.1
LC	131	131	1	-4.3	0.0
LC	132	138	2	-2.7	-0.1
LC	134	141	2	-7.7	NDslow
LC	141	141	1	-7.7	NDslow
LC	144	148	2	-6.2	0.2
LC	144	149	2	-2.3	-0.2
LC	144	151	3	-2.9	0.0
LC	144	154	3	-2.1	-0.2
LC	149	149	0	-3.5	-0.4
LC	150	151	0	-5.3	0.2

LC	151	157	3	-4.9	-0.1
LC	152	153	0	0.4	0.0
LC	152	157	3	-4.7	-0.1
LC	154	155	0	-4.8	-0.1
LC	154	157	2	-5.1	0.0
LC	156	157	1	-5.3	0.0
LC	157	159	0	-3.7	-0.2
LC	157	167	2	-3.0	-0.2
LC	160	167	2	-3.1	-0.2
LC	160	178	2	-1.0	-0.2
LC	170	181	2	-4.0	-0.2
LC	170	184	2	-5.2	0.0
LC	170	185	2	-6.9	0.1
LC	181	184	1	-7.7	NDslow
LC	184	187	1	-6.1	0.0
LC	187	189	1	-2.0	0.1
LC	187	191	2	-2.9	-0.1
LC	188	191	1	-1.6	-0.1
LC	193	193	1	-6.4	-0.1
LC	194	198	2	-7.1	0.1
LC	194	201	3	-7.7	NDslow
LC	199	201	0	-7.7	NDslow
LC	204	207	0	-1.8	0.0
LC	204	215	3	-3.6	0.0
LC	204	220	4	-2.2	0.0
LC	208	210	1	-2.5	-0.4
LC	208	213	2	-3.4	0.0
LC	208	220	3	-2.7	0.1
LC	210	213	1	-3.5	0.0
LC	210	220	2	-2.4	0.0
LC	212	220	2	-6.3	0.3
LC	213	215	0	-6.8	0.0
LC	213	220	2	-7.7	NDslow
LC	216	220	2	-2.3	-0.2

ΔG is folding free energy of WT mAb of each segment (corresponding to Figure S8). $\Delta\Delta G$ is the difference in free energy change between WT mAb and JAWA mutant mAb of each segment (corresponding to Figure 4a). NDslow indicates the segment exchanged too slow to determine the $\Delta\Delta G$. White lines are the segments used in Figures 4 and S8. Yellow lines are backups and correspond to the yellow panels in Figure S8.

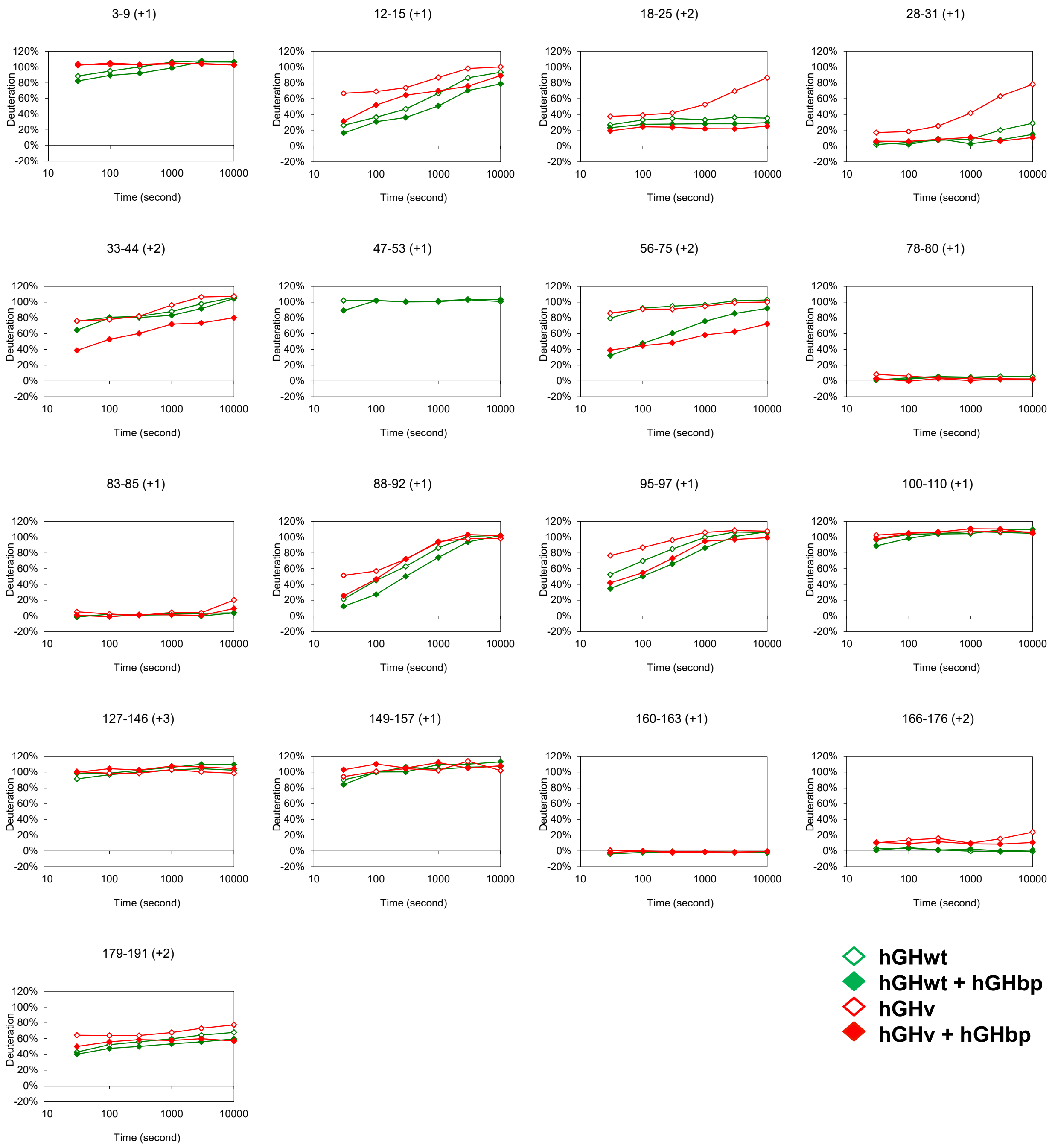

Figure S1. Deuterium incorporation of hGHwt and hGHv with or without hGHbp. Each panel represents a segment of the protein. The title shows the residue numbers in each segment. The number in the parenthesis is the charge state of the peptide analyzed.

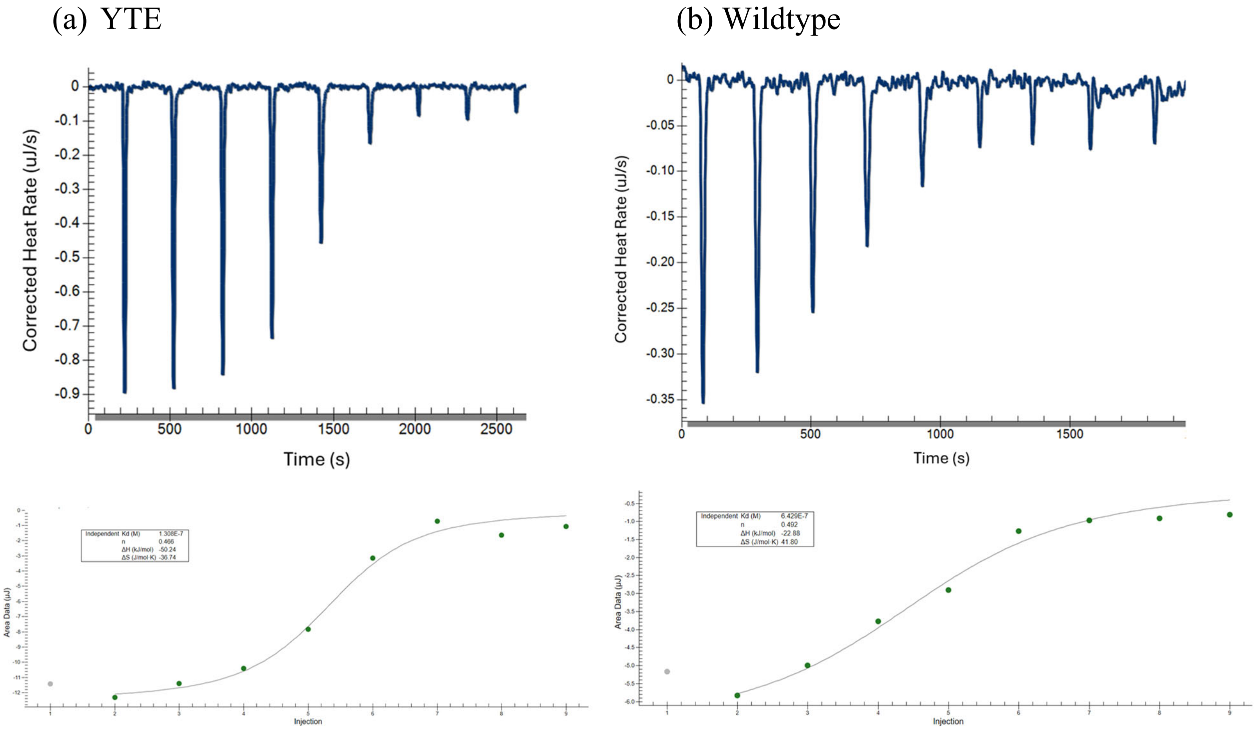

Figure S2. ITC of (a) YTE mutant and (b) wildtype against FcRn. Left panel, the integrated peaks are represented by a fitted curve. Right panel, the raw data with integrated baseline.

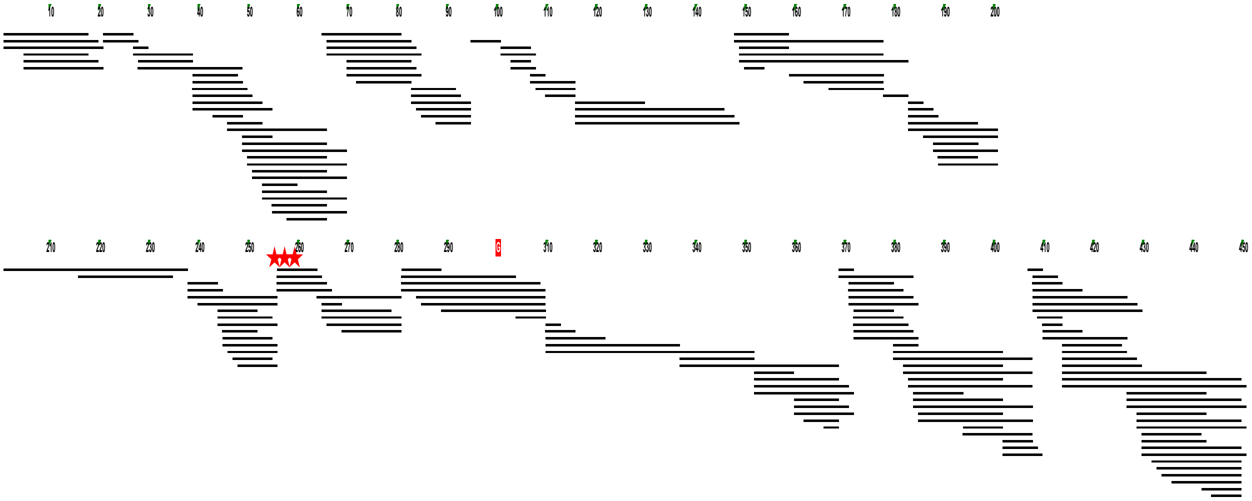

Figure S3. A peptide map of WT and YTE mutant Fc heavy chain digested by pepsin / FPXIII mixed bed column after quenched with 8 M urea, 1 M TCEP, pH 3.0. **G** indicates glycosylation site. **Red** stars are YTE mutation sites.

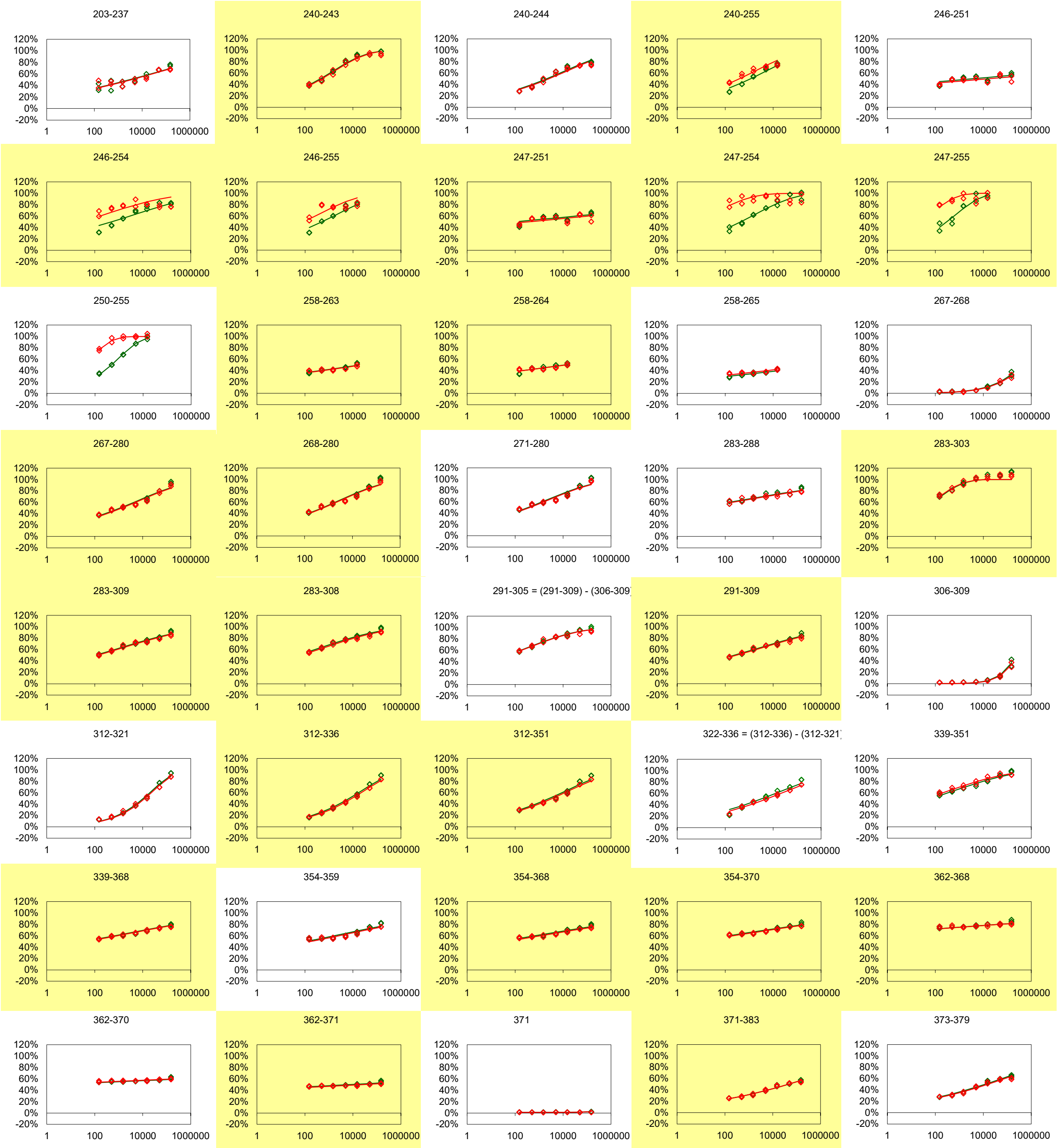

Figure S4. HDX-MS results of WT and YTE mutant heavy chains. Each panel represents deuterium buildup curves of each segment: **Green**, WT; and **red**, YTE. All exchange times are converted to those at pH 7 at 23 °C. White panels are the segments used in the analysis. Yellow panels are backups.

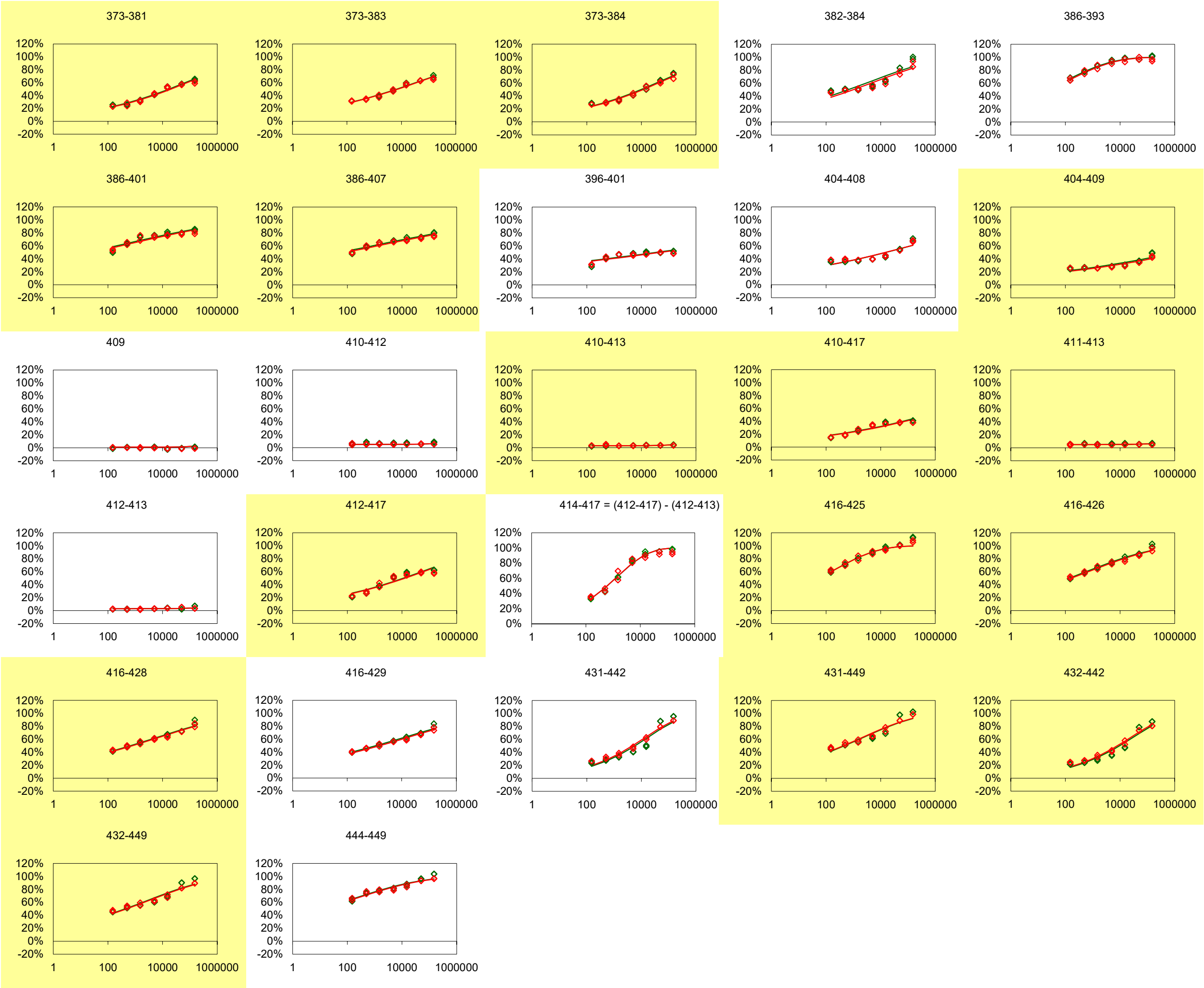

Figure S4 (Continued).

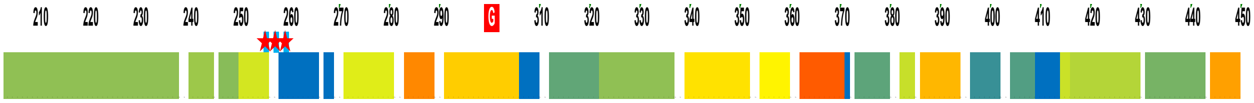

Figure S5. Folding free energy of WT mAb Fc. Reddish color indicates unstable residue (usually not hydrogen bonded) and blueish color indicates stable residue (usually hydrogen bonded). The residues without color indicate the HDX behaviors were not monitored, because there is no peptide to cover the residues or the residues are the first two residues of a peptide. G indicates glycosylation site. **Red** stars are YTE mutation sites.

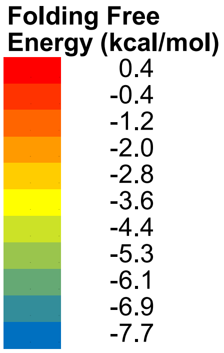

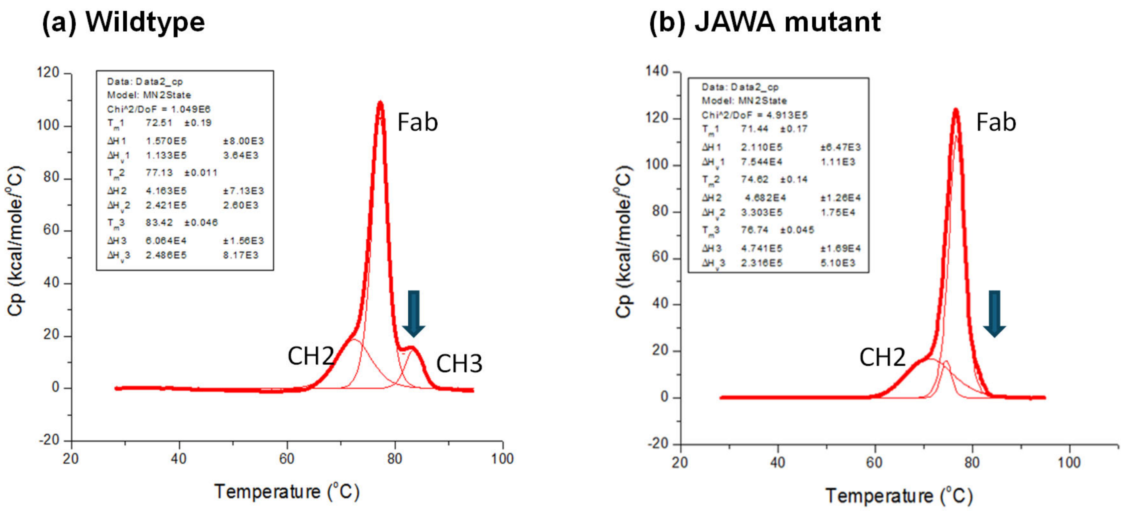

Figure S6. DSC of WT mAb and JAWA mutant mAb.

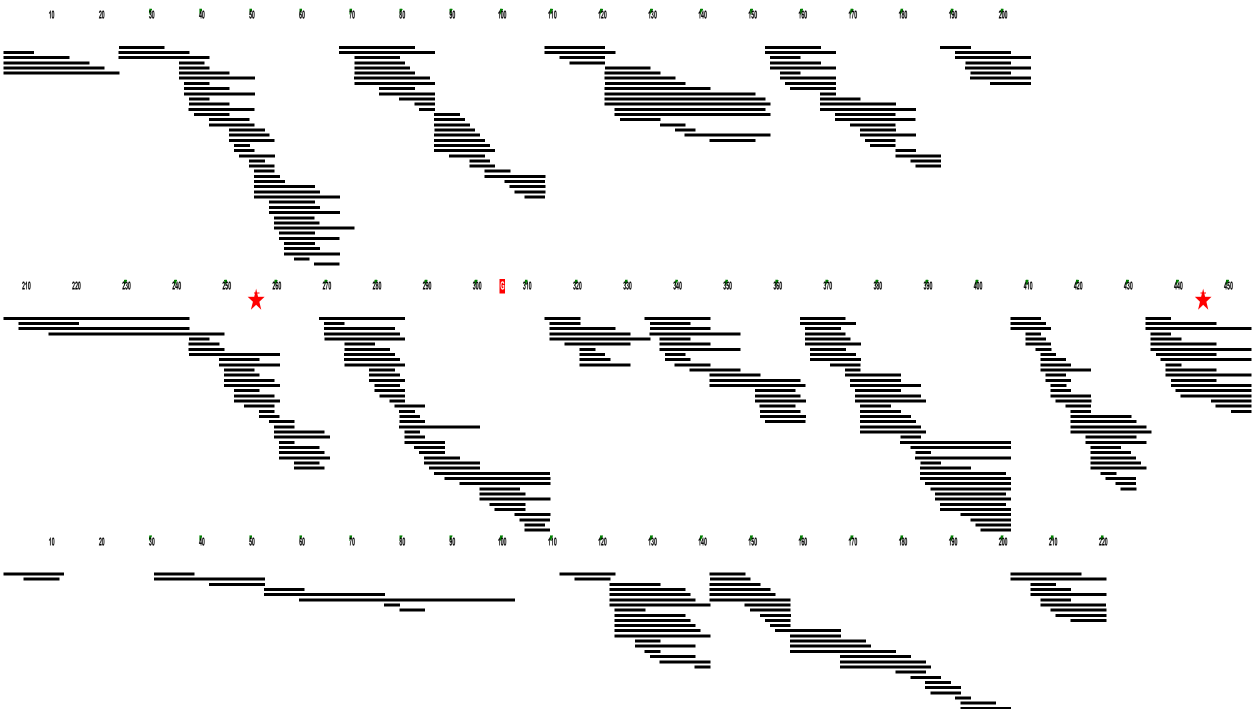

Figure S7. A peptide map of WT mAb and JAWA mutant mAb digested by pepsin / FPXIII mixed bed column after quenched with 8 M urea, 1 M TCEP, pH 3.0. G indicates glycosylation site. **Red** stars are JAWA mutation sites.

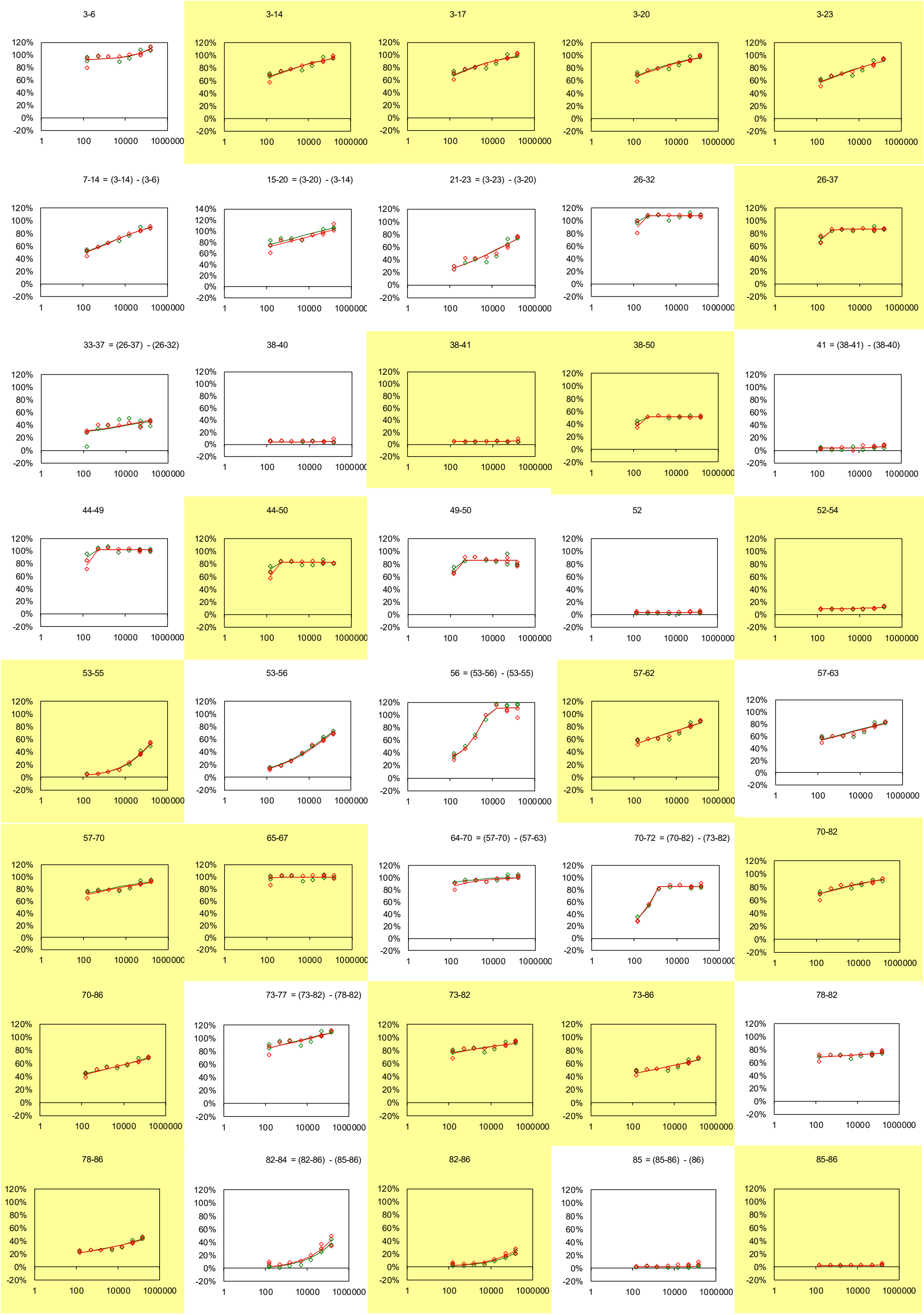

Figure S8a. HDX-MS results of WT and JAWA mutant heavy chains. Each panel represents deuterium buildup curves of WT and JAWA mutant segment: **Green**, WT; **red**, JAWA mutant. All exchange times are converted to those at pH 7 at 23 °C. White panels are the segments used in the analysis. Yellow panels are backups.

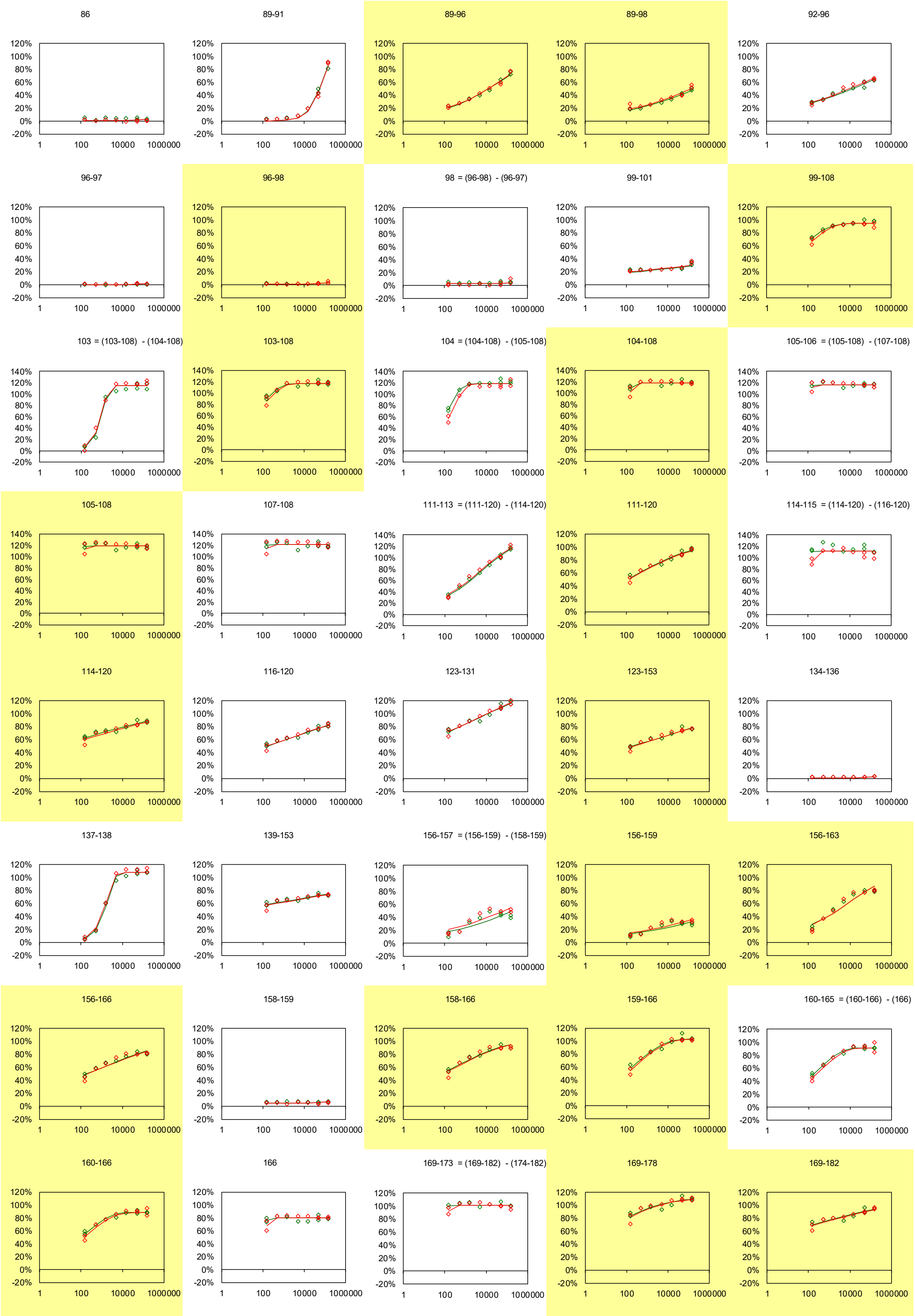

Figure S8a (Continued)**.**

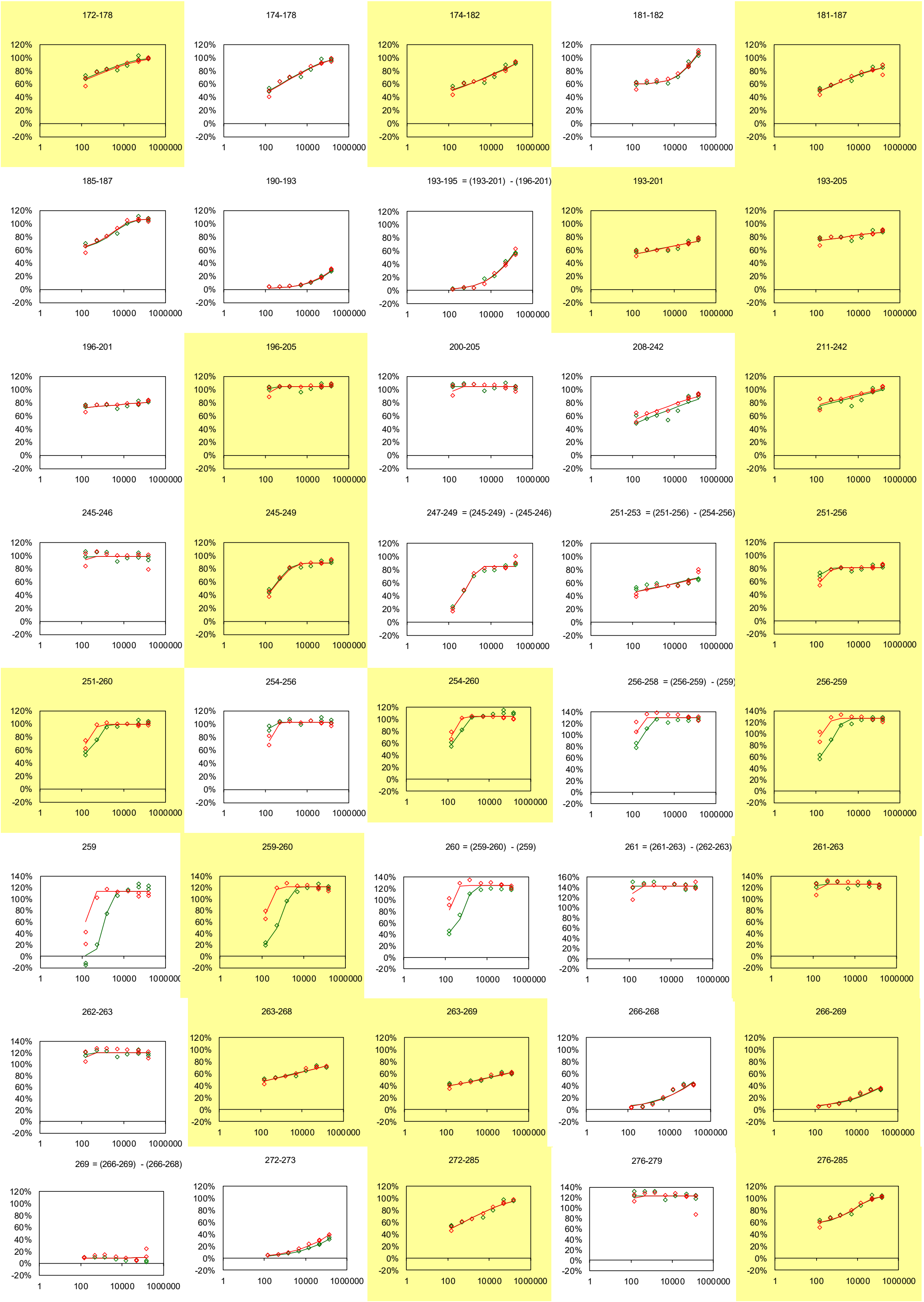

Figure S8a (Continued).

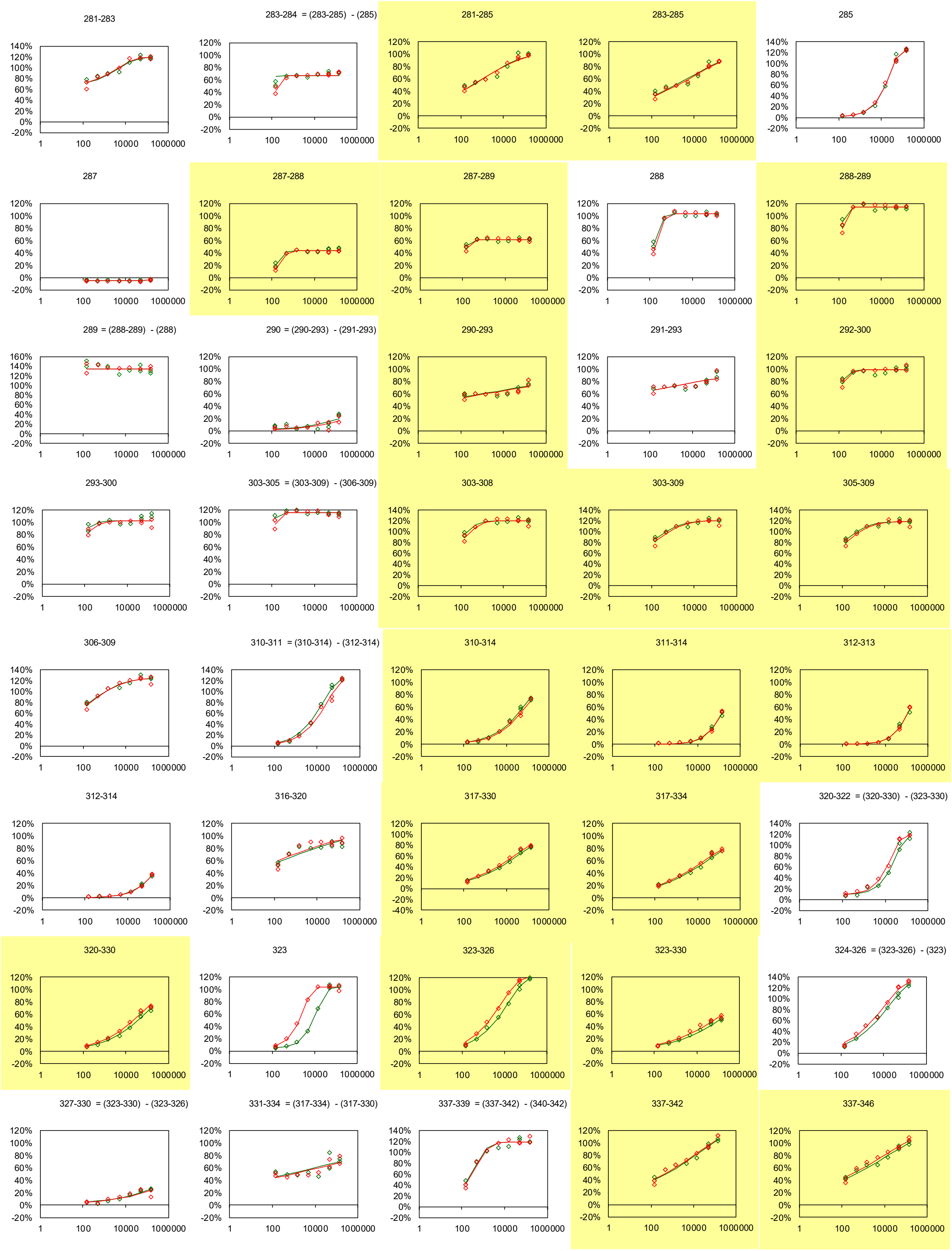

Figure S8a (Continued).

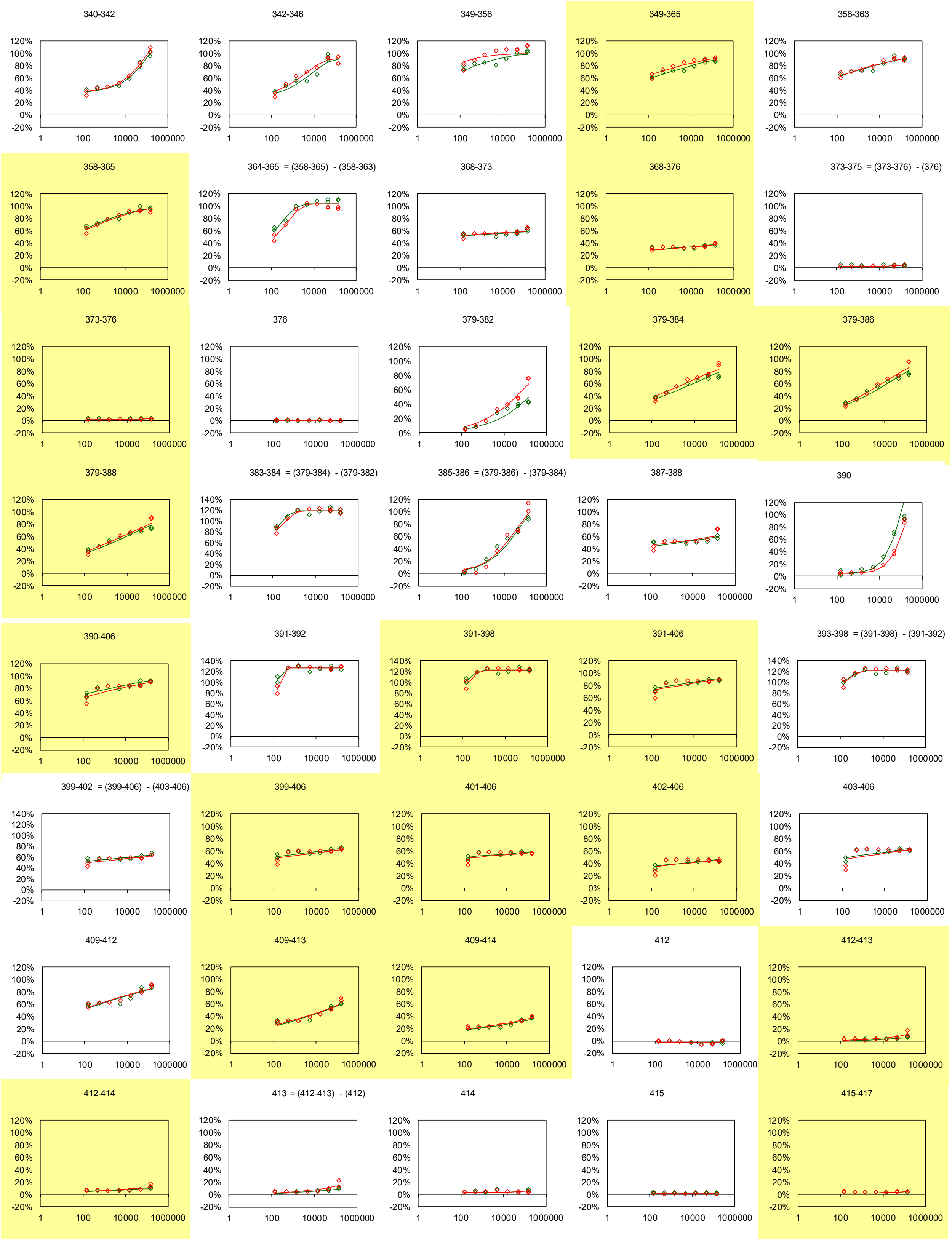

Figure S8a (Continued).

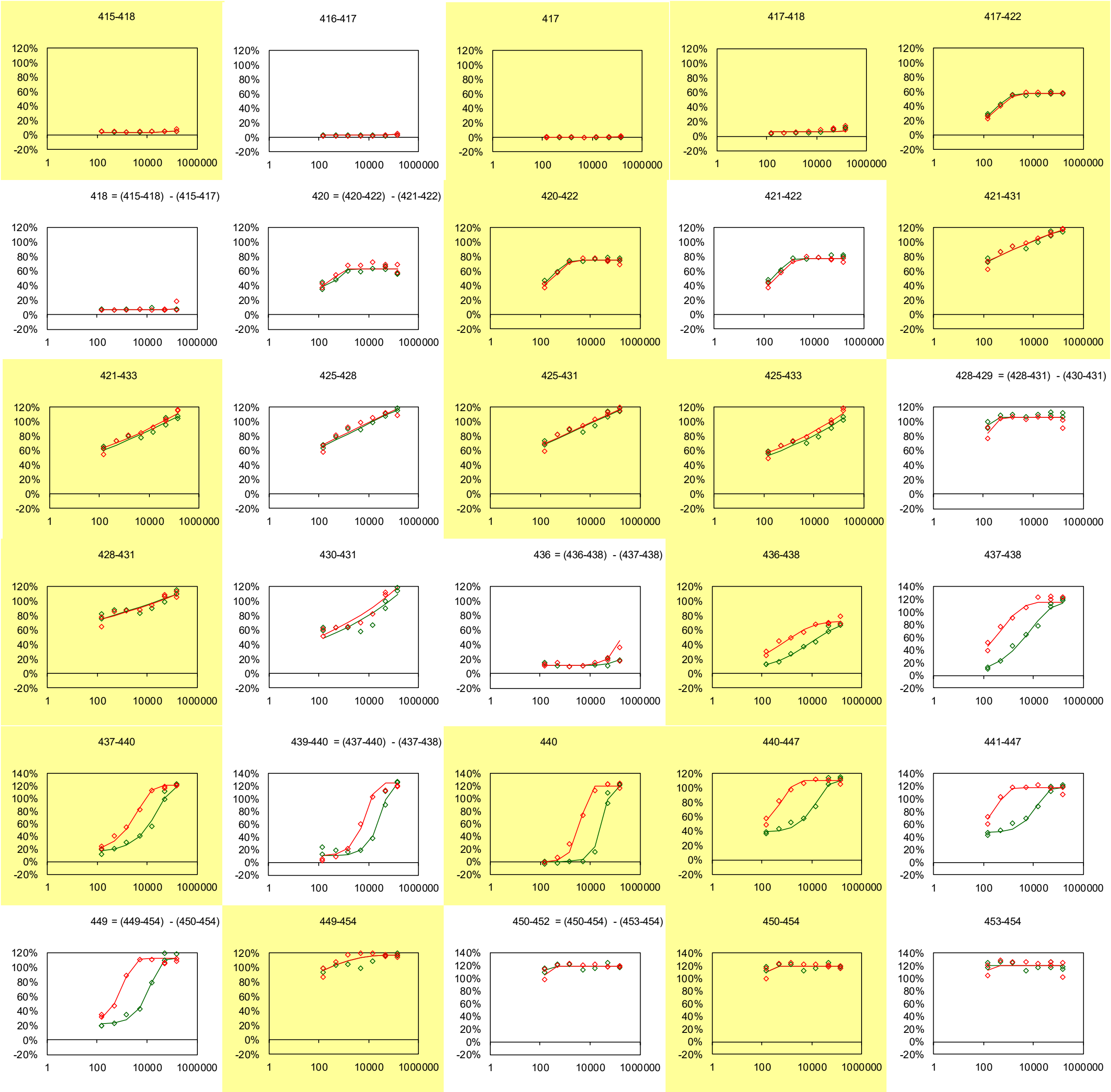

Figure S8a (Continued).

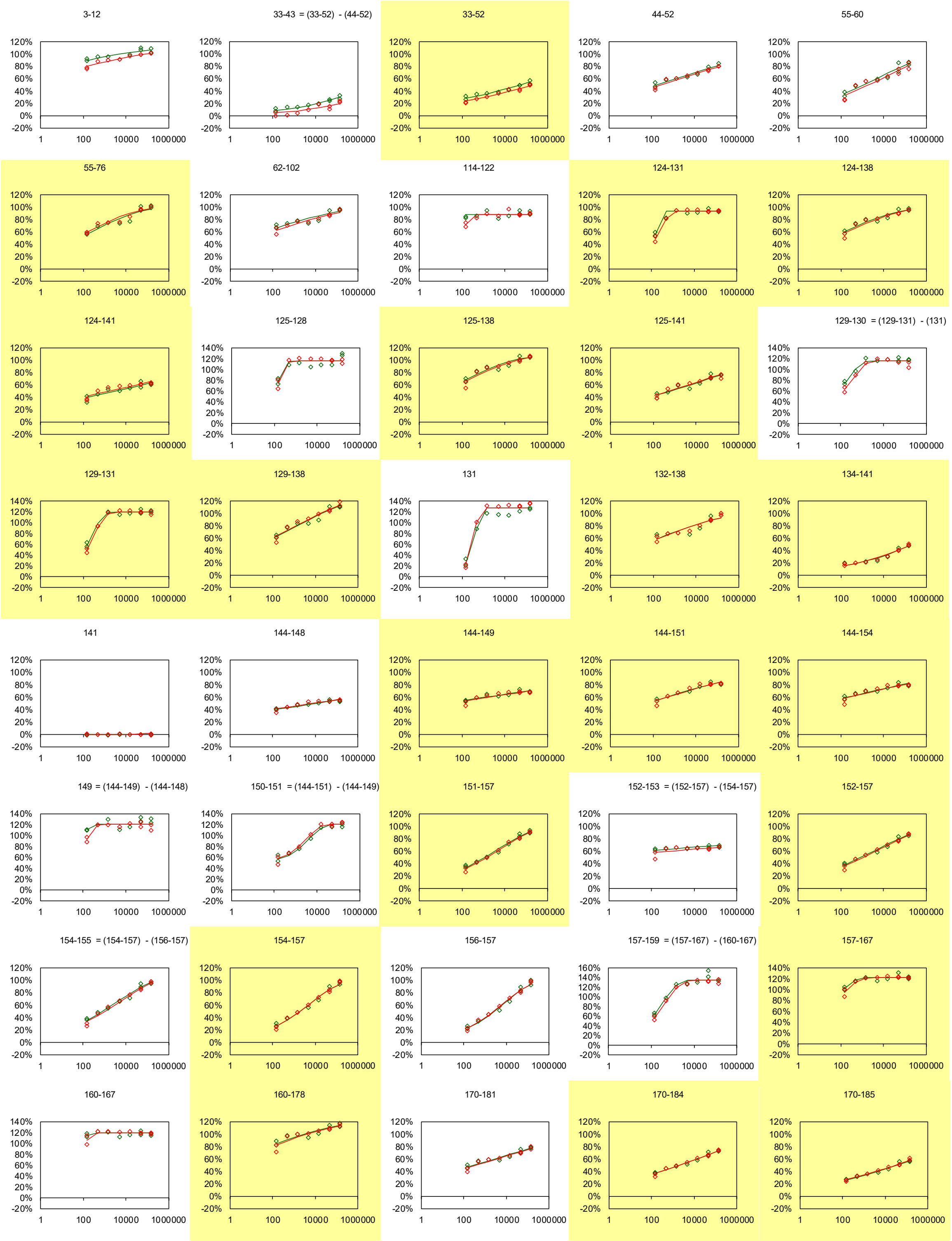

Figure S8b. HDX-MS results of WT and JAWA mutant light chains. Each panel represents deuterium buildup curves of WT and JAWA mutant segment: **Green**, WT; **red**, JAWA mutant. All exchange times are converted to those at pH 7 at 23 °C. White panels are the segments used in the analysis. Yellow panels are backups.

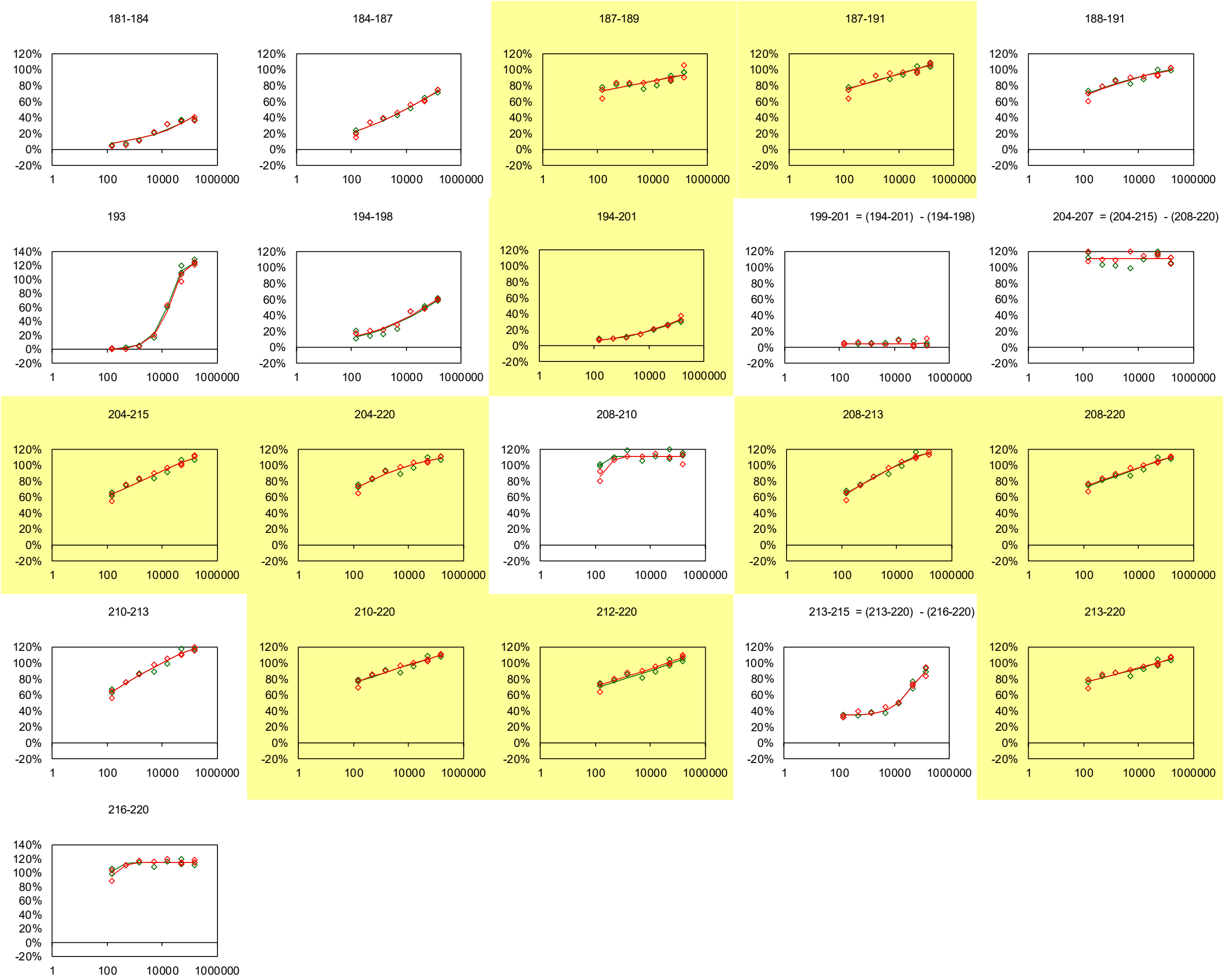

Figure S8b (Continued).

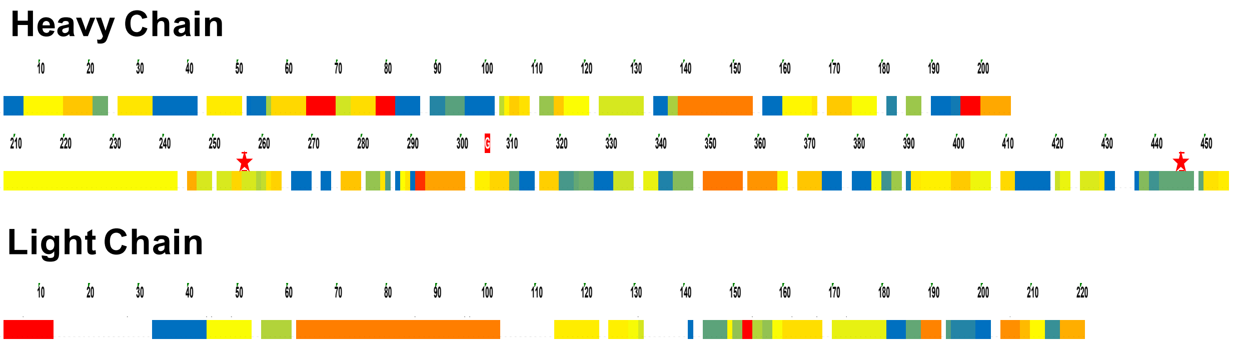

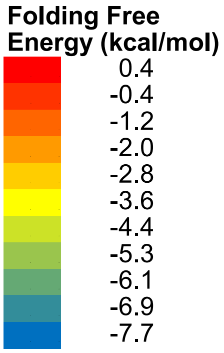

Figure S9. Folding free energy of WT mAb. Reddish color indicates unstable residue (usually not hydrogen bonded) and blueish color indicates stable residue (usually hydrogen bonded). The residues without color indicate the HDX behaviors were not monitored, because there is no peptide to cover the residues or the residues are the first two residues of a peptide. **G** indicates glycosylation site. **Red** stars are JAWA mutation sites.
